# Pierre Teilhard de Chardin: a visionary in controversy

**DOI:** 10.1007/s40656-021-00475-7

**Published:** 2021-11-26

**Authors:** Clément Vidal

**Affiliations:** Center Leo Apostel, Vrije Universiteit Brussel, Krijgskundestraat 33, 1160 Brussels, Belgium

**Keywords:** Noosphere, Cosmic evolution, Cultural evolution, Technological evolution, Direction of evolution, Major evolutionary transition

## Abstract

Teilhard de Chardin developed an evolutionary vision of our planetary future, currently developing from a sphere of life, or *biosphere* towards a sphere of mind, or *noosphere*. As a visionary, Teilhard was not only on the brink of formulating the internet, but he also anticipated current academic efforts to understand globalization, as well as human, cultural and technological evolution. However, his ideas are sources of enduring controversies in both scientific and theological circles. Here I uncover some of the core reasons why his ways of thinking and writing are often problematic, and propose a way forward. This note aims to introduce Teilhard’s central article about the noosphere (*The Formation of the Noosphere*, 1947), but can also be read as an independent introduction to Teilhard’s system of thought. A detailed exegesis of Teilhard’s article is available as a supplementary document.

**Supplementary Information:**

The online version contains supplementary material available at 10.1007/s40656-021-00475-7.

The farther backward you can look, the farther forward you can see.

Winston Churchill.

As a palaeontologist, Pierre Teilhard de Chardin (1881–1955) was comfortable embracing large evolutionary time scales. This is natural for the science of palaeontology, but Teilhard went further in his attempt to foresee the future across a similar evolutionary sweep. Teilhard introduced a new -and often controversial- cosmological vision where evolution is not random, but has a direction towards increasing complexity and consciousness. Centrally, he argued that the next evolutionary stage would be of a planetary nature, a sphere of thinking emerging out of the biosphere that he called the *noosphere*.

Teilhard writes on the brink of the great acceleration that began in the 1950s (Steffen et al., 2015). This acceleration is leading to major planetary changes and we do need global frameworks for dealing with them, as current discourses on our planetary future fail short. The *Anthropocene* discourse focuses on problems and the negative impact of humans (e.g. Shoshitaishvili, 2020). *Globalization* discourse is concerned with socio-political and economic issues, and has troubles caring about and integrating ongoing geosphere and biosphere challenges (e.g. Odum, 2001). The *Gaia hypothesis* (e.g. Lovelock, 1979) takes an organic view of planet Earth but neglects or sees in a negative light human activities and technologies. The *techno*-s*ingularity* discourse (e.g. Kurzweil, 2005) focuses on artificial intelligence, machines, but is rather silent on issues regarding the geosphere or the biosphere. For many, such a techno-utopia is also seen as a dystopia for humanity where AI and robots take over (e.g. Joy, 2000).

By contrast, the noosphere discourse proposes a meaningful vision for the future, where the geosphere, the biosphere and the noosphere -including humans and machines- can work in concert to unleash a new level in evolution. Big historian David Christian (2017) has recently argued that the noosphere idea deserves a second chance. Indeed, the noosphere discourse offers a path toward a positive future, in the sense that it attempts to include in its consideration not only the geosphere and the biosphere, but also to integrate them with human beings, activities, societies and technologies.

My aim is to update and critically analyse the noosphere idea by weaving Teilhard’s insights with contemporary scientific and academic knowledge. To do so, I propose to start with an in-depth analysis of a foundational text on the noosphere, written by Teilhard in 1947: *The Formation of the Noosphere: A Plausible Biological Interpretation of Human History* (translated in Teilhard de Chardin, 1959a). This particular text is often recommended as a first entry into the work of Teilhard (e.g. Steinhart, 2008), not only because of its articulation of the noosphere, but also because it largely avoids the delicate and controversial theme that preoccupied Teilhard in other writings, the synthesis between science and religion.

The text is dense and sometimes difficult, so it needs precise unpacking and updating. Rather than to selectively comment on certain aspects, in a companion document I conduct a systematic exegesis of Teilhard’s essay in the form of a conversation (see supplementary material in this issue). The methodology I follow is *scholarly skywriting* (Harnad, 1990) which combines the advantages of both oral and written communication. It is a communication method that has become natural with the rise of email conversations. As Steven Harnad (2000) noted, “it is also possible in the online medium to make a piece of skywriting come "alive," even if its author is deceased, and to interact with it using all the online dialogic resources for which our brains are specially adapted.”

As a way to introduce my engagement with Teilhard’s essay, in this note I aim to give a modern overview of its key ideas, both the *visionary* and the *controversial* ones. The visionary ones include universal Darwinism, cosmic evolution, human, cultural and technological evolution as well as the noosphere as a superorganism. The more controversial terrain includes his very writing style and conception of science, as well as his law of complexity-consciousness and the assumption that evolution has a direction.

## Teilhard as an evolutionary visionary

### Universal Darwinism

Teilhard can be seen as a pioneer of *universal Darwinism* (Campbell, 2011; Cziko, 1995; Dawkins, 1983), not in the sense of insisting on Darwinian mechanisms, but rather on the universal role and applicability of evolutionary science. He famously wrote (Teilhard de Chardin, 1959b, 219) that evolution is a “general condition to which all other theories, all hypotheses, all systems must bow and which they must satisfy henceforward if they are to be thinkable and true. Evolution is a light illuminating all facts, a curve that all lines must follow”, which also inspired the famous article by his admiring colleague Dobzhansky (1973): *Nothing in Biology Makes Sense Except in the Light of Evolution*. Even today the mainstream evolutionary picture and scope remains much narrower than Teilhard’s, focusing on biological evolution, with a particular focus on genetic evolution (for a history why, see Ruse, 1996). The full potential of the evolutionary worldview remains to be unleashed (see e.g. Wilson, 2019).

### Cosmic evolution

This broad evolutionary vision made Teilhard see *cosmic evolution* in a single sweep, from energy, matter and particles, to chemistry, life, culture and technology. This standard master narrative at the foundation of all sciences is so natural today that it’s hard to appreciate that Teilhard was one of the very first thinkers to fully embrace and argue for the unity of cosmic evolution (Dick, 2009).

Nowadays, some historians have adapted the scientific cosmic evolutionary perspective within their own discipline and developed the field of inquiry and education called “Big History” (e.g. Christian, 2004; Spier, 1996), while the more inspirational and literary facet of this vision is often called the “epic of evolution” (e.g. Rue, 2000).

As a side note, cosmic “evolution” is more accurately a cosmic *development* since there is no evidence of a population of competing universes (Smolin et al., 1997). Teilhard championed such a developmental view, often through biological analogies applied to the universe. Today, assessing in how far cosmic outcomes such as stars, galaxies, life or intelligence are contingent or inevitable developments is at the forefront of research at the intersection of cosmology and complexity science (Vidal, 2010).

### Human, cultural and technological evolution

Teilhard has been a key contributor in the field of human evolution, especially with the discovery of Peking Man (*Homo erectus*) (see e.g. Aczel, 2007). Teilhard’s essay emphasizes the extension of evolution to culture, which is now formally studied as *cultural evolution*. This is remarkable because, at the time of Teilhard’s writing, evolutionary scientists were focused on establishing gene-centric evolution. Cultural evolution and dual inheritance theories were anticipated by other scholars such as David Ritchie (1895) but formal cultural evolutionary modeling would only start in the 1980s (e.g. Boyd & Richerson, 1985). At least as remarkable as Teilhard’s attention to cultural evolution is his emphasis on *technological evolution*. As the reader will discover in my detailed exegesis of the article (see supplementary material), Teilhard had identified the two revolutionary ingredients of what would become the internet: computers and networks. However, he did not take the final step of combining them. Today, it is obvious that technological evolution is developing at a breathtaking speed and is clearly central in our present planetary transition (e.g. Kelly, 2010). Teilhard also sees the rise of *globalization* in terms of a growing circulation of goods and information, but rather uses the word “planetization” highlighting a richer, broader, and more inspiring vision than “globalization” that often emphasizes only the politico-economical sides.

### The noosphere as a superorganism

Teilhard saw human cultural and technological evolution as leading to the formation of the noosphere. He developed this idea together with Édouard Le Roy (1928) and in parallel with Vladimir Vernadsky (1945), but Teilhard emphasized the noosphere's dual meaning: first, it is a sphere of mind like the Greek etymology of the word suggests (*noos*/mind, *sphaira*/sphere), but the noosphere is also argued to be a *superorganism* in formation (this dual meaning was highlighted by Julian Huxley in his introduction to Teilhard de Chardin, 1959b, 13–14). Actually, the organic metaphor and organicist language were foundational for the birth of sociology as a discipline (Barberis, 2003), and Teilhard must have been influenced by this trend while aiming to extend it to the planet as a whole.

To update this idea leading to a planetary transformation, a key concept of modern evolutionary theory is needed: *Major Evolutionary Transitions* (METs). MET theory studies the few transformations in the history of life that lead to major qualitative changes. These include the emergence of the origin of life itself, eukaryote cells, multicellular organisms, sexual reproduction, cultural transmission, mental modelling, and, as a growing number of evolutionary scientists are recognizing and debating, the emergence of a kind of planetary superorganism (Stewart, 2020). While Teilhard kept his focus on providing a *vision* for an upcoming global transition, modern evolutionary scientists are now theorizing the *mechanisms* of METs (e.g. Gillings et al., 2016; Maynard Smith & Szathmáry, 1995). The proposed mechanisms, such as multi-level selectionist logic (e.g. Wilson & Sober, 1994) must address the conditions for and obstacles to the evolution of cooperation at larger and larger scales.

## Teilhard as a controversial figure

If Teilhard is often seen as a highly inspirational figure in popular circles, he is also considered as a highly controversial one in academic theological and scientific circles. Let us discuss why.

### Writing style

From an orthodox scientific perspective his style has been qualified as “bombastic” (Medawar, 1961), or more generally as using freely “poetic metaphors and analogies that are sometimes placed in contexts which represent them as proofs and often extended beyond their original application” (Ayala, 1972, 207). On a yet closer analysis, Teilhard, in his effort to reach a wide audience, mixes many different kinds of discourse unsystematically, from the biological to the metaphysical, theological, and mystical (see e.g. Toulmin, 1982; Glick, 2009). Of course, this writing strategy is a double-edged sword: it can inspire a wide audience (popular, religious, interdisciplinary) but does not always comply with the scientific standards of analytical, empirical knowledge creation. Moreover, his evolutionary theological interpretations threaten traditional theology. As Toulmin (1982, 113) summarises “the theologians among his admirers describe him as a brilliant scientist, the scientists speak of him as a great seer or prophet”.

One space where Teilhard is acknowledged for his influence is in the New Age movement (Lane, 1996). Needless to say, one should not erroneously conclude that because New Age movements use Teilhard’s vision that Teilhard was *only* a new age thinking pioneer.

Some readers might also be at unease with Teilhard’s sturdy optimism. We have to be reminded that Teilhard lived through the two World Wars, and may have felt that he had to provide hope in one of the darkest times of human history. He does so by setting violence, disruptions and suffering in a broader evolutionary context, which can risk minimizing and downplaying them. My point here is that such a tone is likely to appeal more to optimistic personality types, and maybe less to pessimistic types.

### Conception of science

Teilhard was a holistic thinker. He had a very wide conception of science, attempting to understand the totality of phenomena, as opposed to just laws. This comes from the influence that Pierre Duhem’s epistemology had on his early thinking (O’Connell, 1982; Teilhard de Chardin, 1905). As O’Connell argues, Teilhard has a particular methodological take on the way he does science, with a continuous effort to unite the diverse sciences in what Teilhard calls “hyperphysics”. This focus beyond traditional causal mechanisms may partly explain why his work was never fully adopted by the scientific community (see also Toulmin, 1982), and why he is still misunderstood. One can also note that modern epistemology was being developed at the time of Teilhard, for example Popper’s classic opus was published only in (1959).

### Law of complexity-consciousness

It is thus a bit surprising that one of the core elements of Teilhard’s system is the “law of complexity-consciousness” (see especially Teilhard de Chardin, 1966a) because, to qualify as a “law”, a scientific model must be quite robust, fruitful, and universal. But the intuition -if not a law- of increasing complexity and consciousness makes a lot of sense for any thinker who contemplates deep time scales.

A modern way to re-interpret and update Teilhard’s law is to use Bennett’s mathematical concept of logical depth which defines an object as complex if it takes a long computation to generate it (Bennett, 1988; Steinhart, 2008). Cosmic evolution then becomes a kind of decompression (Delahaye & Vidal, 2018) and the phenomenon of science may be interpreted as the beginning of a compression phenomenon resulting in scientific models. This interpretation may also be fruitful for dealing with Teilhard’s theory of the “Omega Point”, but this is outside the scope of this note.

Regarding the consciousness side, one must be reminded that Teilhard is a panpsychist, i.e. the now scientifically marginal idea that all material things have some degree of mentality. As I argue in the companion exegesis supplementary material, there are ways to argue that complexity grows in tandem with consciousness, using central cybernetic results such as the *law of requisite variety* or the *good regulator theorem*.

In this context it is insightful to compare Teilhard and his influential older colleague Henri Bergson, whose books he read. They both defended a version of vitalism as a way to counter purely mechanistic views of the universe. Bergson developed a divergent evolutionary view, where more *novelty* appears in evolution, while Teilhard’s view focused on integration and *convergence* towards an omega point (Weierich, 1971). As Weierich argues, Bergson’s system still has a dualist metaphysics with matter and life, while Teilhard attempts to abolish it with the law of complexity-consciousness. In this way, Teilhard ends with a monist metaphysics where matter has some primitive form of consciousness that can only grow through cosmic evolution.

### Direction of evolution

The law of complexity-consciousness goes hand in hand with the idea of a direction in evolution. This is a difficult and controversial subject-matter, with ramifications down to the epistemological roots of the scientific enterprise itself, involving the concepts of causality and teleology. The main problem from a modern science perspective is that Teilhard’s vitalism implies a grand teleology, a pull towards a future state. This is at the opposite of the requirements to explain everything in terms of simple causality, and thus is vehemently rejected by modern scientific standards. However, this move throws the baby out with the bathwater. Indeed, rejecting any language of goal-directness as non-scientific is a fundamental mistake, at least because it is ignoring the field of cybernetics as well as the attractor concept at the heart of dynamical systems theories (see also the concept of teleodynamics in Deacon, 2012). For example, teleology can be interpreted in a material and causal way, as purpose controlled by feedback (Rosenblueth et al., 1943), and dynamical systems often do tend towards attractors. Both cybernetics and dynamical systems theory are extremely useful and powerful frameworks for science and engineering.

Focusing on evolution, one does *not* need to accept any teleological logic to recognize the evolutionary complexification that has occurred over billions of years. Such complexification doesn’t depend on a pull from the future, as Teilhard claims. This is clear if one considers human evolution: It was not the noosphere that was magically pulling humans to form bigger groups and new organizations since prehistory. Rather, bigger and better managed human groups were able to outcompete smaller and less well managed ones. This point implies that there can be a direction in evolution, even if there is no grand teleology (understood as a pull from the future). To understand and predict how future evolution will unfold is a huge scientific challenge, and Teilhard’s evolutionary vision remains to be assessed.

Also, the direction doesn’t need to be uniquely towards an Omega Point as Teilhard argues. To illustrate this point, imagine rain falling on the top of a mountain. The precise path of each droplet is unpredictable. Since a mountain has a fractal structure, there is actually an infinity of paths leading downhill. If the water starts falling from the top, it might go down on any side of the mountain. There might also be places of stagnation (lakes of all sizes). But one can know for sure the general direction of water: It will roll down. A fitness landscape in evolution has many more dimensions than a three-dimensional mountain, so it’s very hard for our brains to represent it, but essentially the same argument can be made for evolution as a whole: It is largely unpredictable in its details, but a direction can be identified. The analog of gravitation is not one clear factor, but may involve dynamics of natural selection, cooperation and evolvability resulting in increasing average fitness (see also Heylighen, 1999; Stewart, 2000).

Another key aspect of the discussion of directionality has to do with the space and time scales that we consider. Consider another analogy: a reductionist physicist argues that quantum mechanics is the best physics theory because it occurs at the most fundamental and lowest level. Unfortunately, quantum mechanics can’t predict the direction of a cannonball because that occurs at a larger scale. Yet we *can* predict the direction of a cannonball with the larger-scale theory of Newtonian mechanics. A reductionist evolutionary scientist may similarly argue that we can’t predict the direction of evolution because of random genetic mutations and an unpredictable environment. But as Teilhard emphasized, there seem to be large-scale patterns and trends, and we need broader theories to explain them (see Teilhard de Chardin, 1966b). Up to now, evolutionary science might have been in a similar situation as our reductionist physicist, focusing mostly on one level: genes. A core historical reason for this narrow focus was that, in order to establish evolutionary science as a profession, the architects of the evolutionary synthesis used their academic power to reject discussions of evolutionary direction and progress -although they all personally believed in it! (see Ruse, 1996, 410–55).

## Conclusion: a meaningful and inspiring evolutionary vision

Teilhard was undeniably a pioneer and visionary in thinking universal evolution, suggesting the unity of cosmic evolution and development, as well as anticipating modern evolutionary thinking about human, cultural, technological and planetary evolution. If this can be seen as a good track record of predicting entire scientific fields and efforts that would emerge decades later, one can reasonably hope that his more problematic law of complexity-consciousness or his vision of a directional evolution could also become better studied, updated and maybe eventually accepted. In fact, even his most speculative theory of the Omega Point has been updated—first from a scientific and Christian perspective (Tipler, 1995) and then from a purely computational and cosmological perspective (Deutsch, 1997).

Instead of dismissing Teilhard’s positions as a whole because of some of the various controversies and difficulties surrounding his rather unique and multidimensional kind of thinking and writing, it may be more fruitful to see his core claims as key open problems. Even if one can argue that Teilhard’s treatment is insufficient using modern scientific standards, even if he did not uncover the underlying causal mechanisms of the phenomena he described, and even if he used now outdated theories to tackle them, he has the merit of having opened fundamental issues in an inspiring and thought-provoking manner, which can be translated into vibrant debate and a promising scientific agenda.

In particular, his description of the formation of the noosphere remains an underappreciated evolutionary perspective that promises to help us better comprehend and give meaning to the planetary transition we are all experiencing.

## Supplementary Information

Below is the link to the electronic supplementary material.

Supplementary file1 (DOCX 90 KB)
