# Supplementary material for: Pierre Teilhard de Chardin: a visionary in controversy
Source: Hist Philos Life Sci. 2021 Nov 26;43(4):125. doi: 10.1007/s40656-021-00475-7 (PMC8626383; doi:10.1007/s40656-021-00475-7)
Supplement: Supplementary file 1 — Supplementary file1 (DOCX 90 KB) [file 40656_2021_475_MOESM1_ESM.docx]

Teilhard’s *Formation of the Noosphere*:
an exegesis and update

(supplementary material)

Dr. Clément Vidal
Center Leo Apostel,
Vrije Universiteit Brussel
Krijgskundestraat 33, 1160 Brussels, Belgium
contact@clemvidal.com
ORCID iD: <https://orcid.org/0000-0001-6689-5570>

**Abstract**: This essay is a systematic exegesis of Pierre Teilhard de Chardin’s (1959a) essay *The Formation of the Noosphere: A Plausible Biological Interpretation of Human History* that was first published in 1947. My aim here is to update and critically analyse this text by weaving Teilhard’s insights with contemporary scientific and academic knowledge. I cite and comment on the whole text, leaving nothing out, following a “scholarly skywriting” methodology (Harnad 1990). To ease and contextualize the reading of this detailed analysis, I propose a general introduction to Teilhard’s thinking in a short separate note (Vidal 2021).

**Keywords**: noosphere, superorganism, globalization, scholarly skywriting.

We follow the order of Teilhard’s paper, whose outline is:

[1. Birth and Zoological Structure of the Noosphere 4](#__RefHeading___Toc3111_1280809094)

[2. Anatomy of the Noosphere 8](#__RefHeading___Toc3115_1280809094)

[a The apparatus of heredity 9](#__RefHeading___Toc3329_1280809094)

[b The mechanical apparatus 10](#__RefHeading___Toc3331_1280809094)

[c The cerebral apparatus 12](#__RefHeading___Toc3333_1280809094)

[3. The Physiology of the Noosphere 15](#__RefHeading___Toc3117_1280809094)

[4. The Phases and Future of the Noosphere 19](#__RefHeading___Toc3119_1280809094)

[Conclusion: The Rise of Freedom 26](#__RefHeading___Toc3121_1280809094)

[References 30](#__RefHeading___Toc3123_1280809094)

[Note 1 Note in the *Revue des Questions Scientifique* January, 1947. pp. 7-35 where this essay originally appeared: 'To avoid misunderstanding, it may be well to point out that the general synthesis outlined in these pages makes no claim to replace or to exclude the theological account of human destiny. The description of the Noosphere and its attendant biology, as here propounded, is no more opposed to the Divine Transcendence, to Grace, to the Incarnation, or to the Ultimate Parousia, than is the science of paleontology to the Creation, or of embryology to the First Cause. The reverse is true. To those prepared to follow the author in his thinking, it will be apparent that biology merges into theology, and that the Word made Flesh is to be regarded not as a postulate of science -- which would be, in the nature of things, absurd -- but as something, a mysterious Alpha and Omega, taking its place within the whole plan of the universe, both human and divine.' Pierre Charles, S.J.]

This note from Pierre Charles, S.J, a Belgian Jesuit priest who had as a professor and friend Teilhard de Chardin is obviously a way to protect Teilhard from potential religious critiques. Indeed, this visionary text is founded on the science of its time, and mentions the word “God” only once rather poetically and provocatively, and makes hardly any other direct religious statement. Some experts in fact recommend this very text as an introduction to Teilhard de Chardin (Steinhart 2008 note 4).

Gradually, but by an irresistible process (since and through the work of Auguste Comte, Cournot, Durkheim, Levy-Bruhl and many others) the organic is tending to supersede the juridical approach in the concepts and formulations of sociologists.

Teilhard refers to the more general doctrine of *organicism* that sees the universe as living. The organicist position is often contrasted with a *mechanical* and reductionist position, typical of physics and chemistry, that tries to reduce all phenomena to basic mechanisms. By contrast, organicists emphasize that such reductionism is insufficient, that the whole is more than the sum of the parts, that the whole determines the nature of the parts, that the parts cannot be understood in isolation from the whole, and that the parts are dynamically interrelated and interdependent (see e.g. Phillips 1970). Here in particular Teilhard focuses on the idea of society as an organism and we can trace these roots even further than the authors he mentions, for example with Thomas Hobbes (2011) and his *Leviathan*, Herbert Spencer (1895) or even the ancient Greeks (see also Barberis 2003). In his study of the global brain -a concept almost synonymous with the noosphere-, Heylighen (2005; 2007b) covered some of the history of organicism, while Barrow and Tipler (1986) offer a more general history for the idea of the universe as an organism.

A sense of collectivity, arising in our minds out of the evolutionary sense, has imposed a framework of entirely new dimensions upon all our thinking; so that Mankind has come to present itself to our gaze less and less as a haphazard and extrinsic association of individuals, and increasingly as a biological entity

To understand the growth of humankind towards a “biological entity”, one needs to first understand the growth of human groups, from foraging bands, to farming tribes, chiefdoms, kingdoms, early civilization, modern nations, and finally the noosphere. Teilhard doesn’t give details here on the different stages of human social development, but he was certainly aware of prominent thinkers such as Marx, Spencer or Parsons. Today, this hierarchical growth and complexification of human groups is still a central topic to understand human evolution (e.g. Last 2015; or Boehm 2012 in the context of the evolution of morality). These steps are important to better understand and explain the historical context and logic of the mechanisms leading to the birth of the noosphere.

wherein, in some sort, the proceedings and the necessities of the universe in movement are furthered and achieve their culmination.

I see a potential problem here with the idea of “culmination”. If humanity is the *ultimate* culmination of the universe, then this conclusion would be highly anthropocentric. If humanity is the *current* culmination, then it is more arguable that humans are the most sophisticated, complex and influential living beings on the planet... but we still don’t know about the universe as a whole! Also, the word “culmination” implicitly assumes two ideas. First, that evolution tends towards a unique future, as “culmination” comes from the Latin *culmen* and means quite concretely in geography the summit. Second, if there is a summit, there is something to climb and thus a hierarchy and progress in evolution, intuitions which do not lend themselves to precise scientific definitions.

We feel that the relation between Society and Social Organism is no longer a matter of symbolism but must be treated in realistic terms. But the question then arises as to how, in this shifting of values, this passage from the juridical to the organic, we may correctly apply the analogy. How are we to escape from metaphor without falling into the trap of establishing absurd and oversimplified parallels which would make of the human species no more than a kind of composite, living animal? This is the difficulty which modern sociology encounters.

Drawing explicitly on a (biological) analogy has always been seen as problematic scientifically (see Holyoak and Thagard 1995). Another typical example is the Gaïa hypothesis (Lovelock 1979) and its critiques (see e.g. Tyrrell 2013). However, I do agree with Teilhard: today this kind of analogy can be pursued with scientific rigor in a systems theory framework (e.g. Boulding 1956; de Rosnay 1979). In particular, a subset of systems theory called Living Systems Theory (Miller 1978) can help to make the analogy quite clear and systematic (for an application in morality, see Aunger 2017). In Miller’s framework the noosphere level is called the “supranational system”.

It is with the idea and in the hope of advancing toward a solution of the problem that I here venture, basing my argument on the widest possible zoological and biological grounds, to put forward a coherent view of the "thinking Earth" in which I believe we may find undistorted but yet embodying the corrections required by a change of order, the whole process of Life and of vitalization.

By “change of order”, Teilhard refers to what we now call a *major transition* in evolution (e.g. Maynard Smith and Szathmáry 1995; Aunger 2007a; 2007b; Gillings, Hilbert, and Kemp 2016), or a *metasystem transition* (Turchin 1977; Heylighen 2007b). Each transition represents an evolutionary emergence of a higher level of organization and control. Typical examples include the emergence of eukaryotes, multicellularity, or sexual reproduction. This is an extremely important topic, central for understanding and interpreting the noosphere.

To the natural scientist Mankind offers a profoundly enigmatic object of study. Anatomically, as Linnaeus perceived, Man differs so little from the other higher primates that, in strict terms of the criteria normally applied in zoological classification, his group represents no more than a very small offshoot, certainly far less than an Order, within the framework of the category as a whole. But in "biospherical" terms, if I may be allowed the word, man's place on earth is not only predominant but to a certain extent exclusive among living creatures. The small family of hominids, the last shoot to emerge from the main stem of Evolution, has of itself achieved a degree of expansion equal to, or even greater than, that of the greatest vertebrate layers (reptile or mammal) that ever inhabited the earth.

The enigma of human evolution may be unveiled today by understanding the capacity of humans to form and organize groups with larger and larger populations. The expression “the last shoot to emerge from the main stem of Evolution” as the idea of “culmination” earlier would be controversial to defend today as evolutionary biologists do not put the human species at the centre anymore. The evolutionary tree metaphor is now more often replaced by a circular phylogenic tree (Ciccarelli et al. 2006), where the human species occupies a position visually comparable to bacteria. However, from a complexity perspective this visualization is misleading because a human body is many orders of magnitude more complex than a bacterium. Metaphors and visualizations of the living world such as the vertical or circular tree are contested and significant precisely because they give different importance to different species.

Moreover, at the rate it is going, we can already foresee the day when it will have abolished or domesticated all other forms of animal and even plant life.

Teilhard points to the ideas of species extinction by humankind -which is currently a real issue-, as well as the increasing domestication of the living world, which is progressing much as he predicts here with bioengineering, and ecosystemic engineering -even if we don’t do it very well yet!

a We must first give their place in the mechanism of biological evolution to the special forces released by the psychic phenomenon of hominization;

This is a key insight, and is today often taken as a starting point in distinguishing between purely biological evolution, and *cultural evolution*, before considering their dynamical interaction (Richerson and Boyd 2006). This theme will come back in the text.

b Secondly we must enlarge our approach to encompass the formation, taking place before our eyes and arising out of this factor of hominization, of a particular biological entity such as has never before existed on earth -the growth, outside and above the biosphere [Note 2: This term, invented by Suess, is sometimes interpreted (Vernadsky) in the sense of the "terrestrial zone containing life." I use it here to mean the actual layer of vitalized substance enveloping the earth.]– of an added planetary layer, an envelope of thinking substance, to which, for the sake of convenience and symmetry, I have given the name of the Noosphere.

The noosphere is arguably Teilhard’s central concept and contribution. At Teilhard’s time, Vernadsky (1926; 1945) also proposed and discussed a version of the noosphere in his secular, materialist, and non-vitalist worldview (Vernadsky 2012, 41 translator’s note). So, at least through Vernadsky’s work, the noosphere idea can be clearly separated from Teilhard’s unique synthesis of science and religion. Today, we can appreciate similar ideas in Gregory Stock’s (1993) conception of the superorganism that he calls *Metaman*, Otlet’s (1935) anticipation of the world wide web, Well’s (1938) idea of a world brain, as well as the extensive global brain literature (e.g. Russell 1982; Mayer-Kress and Barczys 1995; de Rosnay 1988; 1979; 2000; Heylighen 2002; 2005; 2007b; 2015; Vidal 2015).

1. The birth (or, what amounts to the same thing, the zoological structure);

This equivalence may be surprising, but here we need to understand “birth” as taking place on evolutionary timescales, which requires us to think about the zoological structure and the deep past from which the noosphere emerges.

2. The anatomy;

3. The physiology;

Another general way to speak of anatomy and physiology is to speak about *structure* and *function*. These are general concepts used to describe complex systems, used both in biology and in systems theory (Heylighen 1999b).

# 1. Birth and Zoological Structure of the Noosphere

I HAVE REFERRED to the almost contradictory aspect which the section "homo" in the order of primates assumes in the eyes of natural scientists: that of a single family suddenly emerging, at the end of the Tertiary era, to achieve the dimensions of a zoological layer in itself. If we are to appreciate this strange phenomenon we must look back over the normal development of living forms before the coming of man. It can be characterized in two words: from its first beginnings it never ceased to be "phyletic" and "dispersive."

The definition of “phyletic” means that which has to do with the formation of species, which has to do with the phylum.

Phyletic in the first place: every species (or group of species) formed a sort of shoot (or phylum) which was obliged to evolve "orthogenetically" along certain prescribed lines (reduction or adaptation of limbs, complication of teeth, increased specialization as carnivores or herbivores, runners, burrowers, swimmers, flyers, etc.);

Teilhard refers and adheres to orthogenesis, a now outdated theory of evolution that ascribe evolution to a built-in trend towards progress and greater perfection. As Mayr (1998, 5) writes: “Population thinking [...] is absent in both Geoffroyism and orthogenesis. [...] Their representatives, however, never make it quite clear whether they believe in genuine change [through time] or merely the unfolding of an immanent potentiality.” This outdated belief in orthogenesis, in addition to vitalism are certainly problematic when attempting to update Teilhard’s evolutionary views.

and secondly dispersive, since the different phyla separated at certain points of proliferation, certain "knots" which we may suppose to be periods of particularly active mutation. [Note 5: Dr. A. Blanc has recently given the name of "lysis" to this phenomenon of the releasing of morphological forces].

Teilhard tries to maintain a tension between the two trends of “phyletic” and “dispersive”. However, the theory of *cosmolys* of Alberto Carlo Blanc (1906-1960) is also a kind of orthogenetic theory that has been proven wrong. As Teilhard (1947) wrote about the famous 1947 Paris Colloquium, he saw a tension and a challenge to integrate macro-evolution as studied in palaeontology with micro-evolution that was being discovered via genetics (Glick 2008, 555–57).

Until the coming of man the pattern of the Tree of Life was always that of a fan, a spread of morphological radiations diverging more and more, each radiation culminating in a new "knot" and breaking into a fan of its own.

This “dispersive” dynamic is a differentiation, a variation of species, and thus a divergent dynamic. Today, one may thus rather speak of “convergent dynamic” to describe what Teilhard calls the “phyletic” dynamic, and “divergent dynamic” to describe the “dispersive” dynamic.

But at the human level a radical change, seemingly due to the spiritual phenomenon of Reflection, overtook this law of development. It is generally accepted that what distinguishes man psychologically from other living creatures is the power acquired by his consciousness of turning in upon itself. The animal knows, it has been said; but only man, among animals, knows that he knows. This faculty has given birth to a host of new attributes in men: freedom of choice, foresight, the ability to plan and to construct, and many others. So much is clear to everyone.

Teilhard emphasizes the importance of reflection -or thinking about thinking- as a trigger for radical change. This is a typical example of a higher level of cognitive control, often discovered when a concept is applied to itself, here the concept of knowledge: man “knows that he knows” writes Teilhard (see e.g. Stewart 2001).

But what has perhaps not been sufficiently noted is that, still by virtue of this power of Reflection, living hominized elements become capable (indeed are under an irresistible compulsion) of drawing close to one another, of communicating, finally of uniting. The centers of consciousness, acquiring autonomy as they emerge into the sphere of reflection, tend to escape from their own phylum, which granulates into a line of individuals. Instead they pass tangentially into a field of attraction which forces one toward another, fiber to fiber, phylum to phylum: with the result that the entire system of zoological radiations which in the ordinary course would have culminated in a knot and a fanning out of new divergent lines, now tends to fold in upon itself.

A way to interpret this insight is to say that the power of Reflection leads to a unifying effect. Indeed, in order to share, humans must first assimilate a symbolic and linguistic communication system shared by the larger social group (e.g. Deacon 1998). This inevitably involves a level of conformity and convergence toward a shared mentality.

An other way to interpret this insight is to say that Teilhard is speaking about cultural evolution, and how radically different it is from biological evolution (Richerson and Boyd 2006). Indeed, culture can transfer information quickly as new ideas and know-how can be transferred within a given generation, via conversations, books, etc. This is in sharp contrast with genetic information that can only be renewed after the death of the current generation, and the arrival of the next generation. In evolution, if one visualizes a tree of life, one speaks of horizontal information transfer for cultural evolution, and vertical information transfer for biological evolution. In cultural evolution, convergence of ideas, trends, can thus happen much faster than in biological evolution. In these two ways, one can indeed argue that “evolution is folding in upon itself”.

In time, with the reflexion of the individual upon himself, there comes an inflexion, then a clustering together of the living shoots, soon to be followed (because of the biological advantage which the group gains by its greater cohesion) by the spread of the living complex thus constituted over the whole surface of the globe.

One could attempt to inform the mechanisms behind Teilhard’s argument, using contemporary selectionist logic and group selection theory. His justification of the advantage of groups is just a parenthesis, but in the selectionist framework, it took long and sophisticated arguments to re-establish group selection after it had been mostly dismissed in mainstream biology in 1966 (Wilson 1983).

The critical point of reflexion for the biological unit becomes the critical point of "inflexion" for the phyla, which in turn becomes the point of "circumflexion" (if I may use the word) for the whole sheaf of inward-folding phyla. Or, if you prefer, the reflective coiling of the individual upon himself leads to the coiling of the phyla upon each other, which in turn leads to the coiling of the whole system about the closed convexity of the celestial body which carries us. Or we may talk in yet other terms of psychic centration, phyletic intertwining and planetary envelopment: three genetically associated occurrences which, taken together, give birth to the Noosphere.

Teilhard speaks of three occurrences leading to the Noosphere. The three of them start with a self-referential movement, at three different scales:

1) With “**reflection**”, Teilhard speaks simply of the emergence of human reflection -thinking about thinking-, which he also calls a psychic centration.

2) With “**inflexion”**, Teilhard means that humans are connecting together as a species, and follow a different evolutionary dynamic than other phyla. He speaks about “phyletic intertwining”, meaning that evolution is not only resulting in diverging phyla, but also in convergence (not in the modern sense of “convergent evolution”, but in a Teilhardian sense), especially with the birth of cultural evolution. One could further interpret this as presaging the coming impact of biotechnologies, with generalized genetic manipulations allowing in principle a much greater mixing of genes -- including human, animal, plant and artificially designed genes-- to be combined and recombined.

3) With “**circumflexion**”, Teilhard coins a neologism finishing by “-ion”, just like reflection and inflexion, to mean that the cohesion of human groups facilitates their spread over the entire planet, and thus that we’re dealing with a planetary phenomenon, a planetary envelopment.

Viewed in this aspect, entirely borne out by experience, the collective human organism which the economists so hazily envisage emerges decisively from the mists of speculation to take its place and assume the brilliance of a clearly defined star of the first magnitude in the zoological sky. Until this point was reached Nature, in her generalized effort of "complexification," to which I shall return later, had failed for lack of suitable material to achieve any grouping of individuals outside the family structure (the termitary, the ant hill, the hive).

Social insects including termites, ants and bees are indeed the largest groups that consist of numerous individuals living together as a cohesive “family” (or as a “superorganism”). They are able to coordinate together via the mechanism of *stigmergy* (e.g. Theraulaz and Bonabeau 1999; Parunak 2006; Heylighen 2007a), using external information markers, such as pheromones in termites, ants or bees.

Teilhard notes that existing social insect superorganisms use a kind of “family structure”. This “family” interpretation is well-founded since all the individual members of an insect colony typically stem from one single queen, and thus all the individuals are extremely similar genetically. In the case of humanity, we have a much wider genetic diversity, so stigmergy would not function as well. One can argue that cultural, social, and linguistic skills are key to contribute making human stigmergy more efficient.

With man, thanks to the extraordinary agglutinative property of thought, it has at last been able to achieve, throughout an entire living group, a total synthesis of which the process is still clearly apparent, if we trouble to look, in the "scaled" structure of the modern human world.

The translation here of “écaillée” by “scaled” needs to be commented on because Teilhard means “écaille” in the sense of the scale of a fish, and not in the sense of size.

Anthropologists, sociologists and historians have long noted, without being very well able to account for it, the enveloping and concretionary nature of the innumerable ethnic and cultural layers whose growth, expansion and rhythmic overlapping endow humanity with its present aspect of extreme variety in unity.

With the expression “extreme variety in unity”, Teilhard highlight the theme of differentiation and integration, which generally act in concert. Today, we understand better these dynamics, and differentiation typically can occur once the suppression of free-riders is effective, i.e. when the cooperation problem is largely solved (Szathmáry 2015; West et al. 2015).

This "bulbary" appearance becomes instantly and luminously clear if, as suggested above, we regard the human group, in zoological terms, as simply a normal sheaf of phyla in which, owing to the emergence of a powerful field of attraction, the fundamental divergent tendency of the evolutionary radiations is overcome by a stronger force inducing them to converge.

Teilhard means that human groups organize themselves together, giving rise to groups of groups, i.e. different layers of groups illustrated by the bulb metaphor. This goes beyond the complexity of social insect colonies that don’t really form colonies of colonies recursively. It is also quite inspiring to see in our species a unique form of evolutionary dynamics.

In present-day mankind, within (as I call it) the Noosphere, we are for the first time able to contemplate, at the very top of the evolutionary tree, the result that can be produced by a synthesis not merely of individuals but of entire zoological shoots.

Teilhard wrote above that the “the entire system of zoological radiations which in the ordinary course would have culminated in a knot and a fanning out of new divergent lines, now tends to fold in upon itself” so he seems to mean that the noosphere has to do with the formation of a superorganism, including not only humans, but also other species and their domestication. However, at other places and in other writings, he emphasizes the human species and does not elaborate much on how this synthesis of “entire zoological shoots” would happen or what it would be like.

Thus we find ourselves in the presence, in actual possession, of the superorganism we have been seeking, of whose existence we were intuitively aware. The collective mankind which the sociologists needed for the furtherance of their speculations and formulations now appears scientifically defined, manifesting itself in its proper time and place, like an object entirely new and yet awaited in the sky of life. It remains for us to observe the world by the light it sheds, which throws into astonishing relief the great ensemble of everyday phenomena with which we have always lived, without perceiving their reality, their immediacy or their vastness.

Teilhard uses the term “superorganism” here. It is interesting that he writes that we possess it. It makes sense from the Noosphere perspective, if it is interpreted as the nervous system of Earth, then the superorganism is controlled by the noosphere. The question of whether and to which degree we control or we will control the superorganism is of course of fundamental importance.

# 2. Anatomy of the Noosphere

IT MAY BE said, speaking in very general terms, that in asserting the zoological nature of the Noosphere we confirm the sociologists' view of human institutions as organic.

There is a mistranslation here. Teilhard’s point is not that human institutions are organic. The french says “la nature zoologique de la Noosphère confirme pleinement les institutions organicistes de la Sociologie”. By this he means that his view of the Noosphere so far confirms the sociologists’ positions of organicism (inside sociological institutions) that he was referring to at the beginning of the chapter. Another translation (avoiding the confusing “institution” word) would be: “IT MAY BE said, speaking in very general terms, that having identified the zoological nature of the Noosphere we fully confirm the organicist’s position in sociology”. This is no small claim, as organicism in sociology had been largely dismissed, even if it influenced greatly the birth of the discipline (Barberis 2003).

Once the exceptional, but fundamentally biological, nature of the collective human complex is accepted, nothing prevents us (provided we take into account the modifications which have occurred in the dimensions in which we are working) from treating as authentic organs the diverse social organisms which have gradually evolved in the course of the history of the human race.

The idea of social organisms that evolve is stimulating, although we would need to specify how they evolve. One could also take a functional view here, rather than an organ-centred view, because the organs tends to be localized spatially, while functions can be localized or *distributed*. For example the function of security, implemented through the immune system of the body, is not localized in any organ.

Directly Mankind, from the nature of its origin, presents itself to our experience as a true superbody, the internal connections of this body, by reason of homogeneity, can only be treated and understood as superorgans and supermembers. Thus, for example (due allowance being made for the change of scale and environment), it becomes legitimate to talk in the sphere of economics of the existence and development of a circulatory or a nutritional system applicable to Mankind as a whole.

Teilhard wants to take the biological analogy strongly here with expressions such as “authentic organs”, “true superbody”, “superorgans and supermembers”. The example of economics actually has two faces. First, the circulation of money is an *information* circulation, and second, the nutritional system is a *matter-energy* distribution system. Systems theorists have understood that the two flows circulate in opposite direction. A flow of money in one direction triggers a flow of matter-energy in the opposite direction (e.g. de Rosnay 1979). This systems theory account of counter flow is an important addition to Teilhard’s discussion of what economics represents in the superorganism.

That we must proceed slowly and critically in this attempt to construct an "anatomy" of society is evident. Used without discernment and a profound knowledge of biology, the procedure is in danger of lapsing into puerile and sterile subtleties. But progressively pursued, and proceeding from certain major fields of knowledge, the method shows itself to be both fruitful and illuminating. This is what I shall seek to demonstrate in the three spheres of culture, machinery and research, by successively "dissecting" first the hereditary, then the mechanical and finally the cerebral apparatus of the Noosphere.

Teilhard is warning against the misuse of analogies and metaphors and announces the structure of this section.

## a The apparatus of heredity

One of the paradoxes attaching to the human species, a cause of some bitterness among biologists, is that every man comes into the world as defenseless, and as incapable of finding his way single-handed in our civilization, as the newborn Sinanthropus a hundred thousand years ago. As Jean Rostand [Note 6: *Pensées d'un biologiste*, pp. 32-35] has remarked, during the many centuries man has striven to improve himself the fruits of his labors have brought about no organic change in him, they have not affected his chromosomes.

This remark is another way to say that Lamarckian evolution doesn’t take place (I mean before the discoveries of epigenetics, somatic hypermutation or the inheritance of the hologenome). One may thus wonder why Teilhard is often said to be Lamarckian. The answer lies in distinguishing the two fundamentals tenets of Lamarckism: one is a complexifying force (to which Teilhard subscribes), the other is an adaptive force of inheritance of acquired characters (to which Teilhard doesn’t subscribe).

So much so, the author goes on to imply, that all the advances on which we so pride ourselves remain biologically precarious, superficial or even exterior to ourselves.

This theme of exteriority is central here, and indeed exteriority enhances and extends cognition (see e.g. Clark and Chalmers 1998; Clark 2003; Vidal 2015) contributing to a fundamentally new dynamic in evolution, namely cultural evolution.

There is much that might be said about this; but let us pass over the question of whether we have not undergone some modification, even in our chromosomes, since the era of the pre-Hominids or even that of Cro-Magnon man. Let us concede provisionally that we have developed no hereditary trait in that period rendering us more innately capable of perception and movement in the new dimensions of society, space and time. How does this affect our appreciation and evaluation of human progress? I shall show that the answer is splendid and highly encouraging-provided we do not lose sight of the organic reality of the Noosphere.

Teilhard is trying to develop a view of humanity that offsets the evolutionary fact that the knowledge accumulated in individual minds is lost at their death. He wants to shift our perspective towards a global, extended view of progress in human evolution.

"Separate the newborn child from human society," you may say, "and you will see how weak he is!" But surely it is clear that this act of isolation is precisely what must not be done, and indeed cannot be done. From the moment when, as I have said, the phyletic strands began to reach toward one another, weaving the first outlines of the Noosphere, a new matrix, coextensive with the whole human group, was formed about the newly born human child -a matrix out of which he cannot be wrenched without incurring mutilation in the most physical core of his biological being.

Teilhard takes the metaphor of a matrix here, hinting a systems understanding while dismissing the analytical thought experiment that would attempt to “separate the newborn child from human society”. This thought experiment was typical in 18th century discussions: for examples Rousseau’s (2002) famous thought experiments about the social contract were designed to present human nature as disconnected from the rest of nature.

Traditions of every kind, hoarded and manifested in gesture and language, in schools, libraries, museums, bodies of law and religion, philosophy and science-everything that accumulates, arranges itself, recurs and adds to itself, becoming the collective memory of the human race-all this we may see as no more than an outer garment, an epiphenomenon precariously superimposed upon all the other edifices of Nature (the only truly organic ones, as it may appear): but it is precisely this optical illusion which we have to overcome if our realism is to reach to the heart of the matter. It is undoubtedly true that before Man hereditary characteristics were transmitted principally through the reproductive cells. But after the coming of Man another kind of heredity shows itself and becomes predominant; one which was indeed foreshadowed and essayed long before Man, among the highest forms of insects and vertebrates [Note 7: A small cynocephalus (baboon), born in captivity, will commit all kinds of blunders when set free (heredity of education). But in similar conditions a young otter, being put in the water, will at once know how to behave (chromosomic heredity). Cf. Eugene N. Marais, *The Soul of the White Ant*.].

This is the heredity of example and education. In Man, as though by a stroke of genius on the part of Life, and in accord with the grand phenomenon of phyletic coiling, heredity, hitherto primarily chromosomic (that is to say, carried by the genes) becomes primarily "Noospheric" -transmitted, that is to say, by the surrounding environment. In this new form, and having lost nothing of its physical reality (indeed, as much superior to its first state as the Noosphere is superior to the simple, isolated phylum) it acquires, by becoming exterior to the individual, an incomparable substance and capacity.

Teilhard articulates what we now call dual inheritance theories, or cultural evolution in addition to biological evolution (see e.g. Boyd and Richerson 1985; Aunger 2000).

For let me put this question: what system of chromosomes would be as capable as our immense educational system of indefinitely storing and infallibly preserving the huge array of truths and systematized technical knowledge which, steadily accumulating, represents the patrimony of mankind? Exteriorization, enrichment: we must not lose sight of these two words. We shall come upon them again, quite unchanged, when we turn to consider the machine.

Teilhard is spot on here on the impact and importance of cultural evolution. Regarding exteriorization and enrichment, I have argued that preserving, augmenting and promoting the rise of organized complexity can be seen as ethical imperatives (Delahaye and Vidal 2019).

## b The mechanical apparatus

The fact was noted long ago:[Note 8: e.g., Edouard Le Roy, *Les Origines Humaines et le Problème de l'intelligence*] what has enabled man zoologically to emerge and triumph upon earth, is that he has avoided the anatomical mechanization of his body. In all other animals we find a tendency, irresistible and clearly apparent, for the living creature to convert into tools, its own limbs, its teeth and even its face. We see paws turned into pincers, paws equipped with hooves for running, burrowing paws and muzzles, winged paws, beaks, tusks and so on-innumerable adaptations giving birth to as many phyla, and each ending in a blind alley of specialization. On this dangerous slope leading to organic imprisonment Man alone has pulled up in time. Having arrived at the tetrapod stage he contrived to stay there without further reducing the versatility of his limbs. Possessing hands as well as intelligence, and being able, in consequence, to devise artificial instruments and multiply them indefinitely without becoming somatically involved, he has succeeded, while increasing and boundlessly extending his mechanical efficiency, in preserving intact his freedom of choice and power of reason.

The significance and biological function of the tool at last separated from the limb has, as I was saying, long been recognized; and it has long been realized that the tool separated from Man develops a kind of autonomous vitality. [Note 9: e.g., Jacques Lafitte, *Réflexions sur la Science de la Machine*. La Nouvelle Journée, no. 21, 1932.] We have passive machines giving birth to the active machine, which in turn is followed by the automatic machine.

Teilhard does not give definitions or examples for what he means by passive, active or automatic machines. One can surmise that a passive machine does not require energy (e.g. a shovel) and that an active machine requires energy (e.g. an engine). By automatic machine he probably means machines with several mechanisms that are linked, such as the computer.

Those are the main classifications; but within each classification what an immense proliferation there is of branches and offshoots, each endowed with a sort of evolutionary potential, irresistible both logically and biologically! We have only to think of the automobile or the airplane. All this has been noted and often said.

But what has not yet been sufficiently taken into account, although it explains everything, is the extent to which this process of mechanization is a collective affair, and the way in which it finally creates, on the periphery of the human race, an organism that is collective in its nature and amplitude.

Teilhard puts emphasis on the role of human collective action and knowledge leading to a planetary organism. This is further accelerated with the rise of technology and machines. Teilhard is extremely prescient here with his vision of the evolutionary potential of technology. For a modern overview see for example (Kelly 2010).

Let us consider this. With and since the coming of Man, as we have seen, a new law of Nature has come into force-that of convergence.

One could question the translation of “rapprochement” by “convergence” here. Indeed, the word “convergence” exists in French, but “rapprochement” means more simply “bringing together”. So “bringing together” would be more suitable, especially because Teilhardian convergence doesn’t have the same meaning as “convergence” in modern evolutionary biology.

The convergence of the phyla both ensues from, and of itself leads to, the coming together of individuals within the peculiarly "attaching" atmosphere created by the phenomenon of Reflexion. And out of this convergence, as I have said, there arises a very real social inheritance, produced by the synthetic recording of human experience. But if we look for it we may observe precisely the same phenomenon in the case of the machine.

The rise and evolution of machines are as important as the phenomenon of reflection, and follow a similar dynamic. This emphasis on machines and their evolutionary potential is again quite remarkable.

Every new tool conceived in the course of history, although it may have been invented in the first place by an individual, has rapidly become the instrument of all men; and not merely by being passed from hand to hand, spreading from one man to his neighbor, but by being adopted corporatively by all men together. What started as an individual creation has been immediately and automatically transformed into a global, quasi-autonomous possession of the entire mass of men. We see this from prehistoric times, and we see it with a vivid clarity in the present era of industrial explosion. Consider the locomotive, the dynamo, the airplane, the cinema, the radio -anything. Can there be any doubt that these innumerable appliances are born and grow, successively and in unison, from roots established in an existing mechanical world-state? For a long time past there have been neither isolated inventors nor machines. To an increasing extent every machine comes into being as a function of every other machine; and, again to an increasing extent, all the machines on earth, taken together, tend to form a single, vast, organized mechanism.

Teilhard develops a systems and networked view of technology and its spread as well as its recursive improvement. Tools and machines cannot be isolated for long. Of course, there are examples of lagging (e.g. gunpower that took time to spread), leapfrogging (e.g. Africa that used massively mobile phones without first using landline phones). But Teilhard has arguably evolutionary timescales in his head, and this may explain why from this high level perspective technological spread seems like almost “immediate”.

Necessarily following the inflexive tendency of the zoological phyla, the mechanical phyla in their turn curve inward in the case of man, thus accelerating and multiplying their own growth and forming a single gigantic network girdling the earth. And the basis, the inventive core of this vast apparatus, what is it if not the thinking center of the Noosphere? When *Homo faber* came into being the first rudimentary tool was born as an appendage of the human body. Today the tool has been transformed into a mechanized envelope (coherent within itself and immensely varied) appertaining to all mankind. From being somatic it has become "noospheric." And just as the individual at the outset was enabled by the tool to preserve and develop his first, elemental psychic potentialities, so today the Noosphere, disgorging the machine from its innermost organic recesses, is capable of, and in process of, developing a brain of its own.

Teilhard emphasizes the importance and impact of externalization of cognition (Clark and Chalmers 1998). Generalizing the movement of the externalization of cognition is a way to think systematically about how to build the noosphere (or global brain) (Vidal 2015).

## c The cerebral apparatus

Between the human brain, with its billions of interconnected nerve cells, and the apparatus of social thought, with its millions of individuals thinking collectively, there is an evident kinship which biologists of the stature of Julian Huxley have not hesitated to examine and expand on critical lines [Note: 10 Lecture delivered in New York and published in the *Scientific Monthly*, 1940]. On the one hand we have a single brain, formed of nervous nuclei, and on the other a Brain of brains.

The brain analogy is attractive indeed, but insufficient on its own to prove the point. Just because two systems both have a huge number of components does not mean that they will be similar on other respects. But, as analogies can be used as heuristics to probe structural and functional similarities, this exercise is certainly worth pursuing (see e.g. Holyoak and Thagard 1995). For example, it has been suggested that a transition of organization and major new features arise when a system increases its number of elements by an order of magnitude. Peter Russell (1995) argues that the world’s population increasing towards 10 billion humans - and I would add, the many more billions of machines that are also increasing - is an example of such a transition, or perhaps even awakening. Of course, the sheer number of components doesn’t guarantee interconnection, interaction, hierarchies, and dynamical control that are some of the hallmarks of complexity.

It is true that between these two organic complexes a major difference exists. Whereas in the case of the individual brain thought emerges from a system of nonthinking nervous fibers, in the case of the collective brain each separate unit is in itself an autonomous center of reflection. If the comparison is to be a just one we must, at every point of resemblance, take this difference into account. But when all allowance is made the fact remains that the analogies between the two systems are so numerous, and so compelling, that reason forbids us to regard the parallel as either purely superficial or a mere matter of chance.

Let us take a closer look at analogical reasoning. First, an analogy can be defined as a structural or functional similarity between two domains of knowledge. The two domains are called the source and the target domains. One can then distinguish different kinds of analogies: *positive*, *negative*, and *neutral* analogies (Hesse 1966, 8). A positive analogy addresses the question ‘‘What is analogous?’’ and constitutes the set of relations that hold in the two domains. A negative analogy addresses the question ‘‘What is disanalogous?’’ and constitutes the set of relations that do not hold in the two domains. Finally, neutral analogies trigger the question: ‘‘Are the two domains analogous?’’ To answer this last question, one has to examine or test whether this or that relation holds in the target domain.

In this case, Teilhard points to one “negative” analogy when considering the brain (source domain) to the noosphere (target domain). The difference is simply that the elements in the brain (the neurons) are not conscious, while the elements in the noosphere (humans) are conscious.

It is worth mentioning how fruitful and impactful an analogy can be, through the story of Tim Berners Lee (1999, 4 & 41), when he founded the world wide web. He reports that he was largely inspired by the free association capabilities of the brain, and wanted to endow computers with analogous capacities for linking together any type of information. This resulted in nothing less than the web. In this perspective, it’s no wonder that the web works a bit like a brain, because it’s founder designed it to function as ... a brain! This is an interesting case where an analogy is strengthened because we humans believe in it and act accordingly.

Let us take a rapid glance at the structure and functioning of what might be termed the "cerebroid" organ of the Noosphere. First the structure: and here I must turn back to the machine. I have said that, thanks to the machine, Man has contrived both severally and collectively to prevent the best of himself from being absorbed in purely physiological and functional uses, as has happened to other animals. But in addition to its protective note, how can we fail to see the machine as playing a constructive part in the creation of a truly collective consciousness? It is not merely a matter of the machine which liberates, relieving both individual and also of the machine which creates, helping to assemble, and to concentrate in the form of an ever more deeply penetrating organism, all the reflective elements upon earth. I am thinking, of course, in the first place of the extraordinary network of radio and television communications which, perhaps anticipating the direct syntonization of brains through the mysterious power of telepathy, already link us all in a sort of "etherized" universal consciousness.

At the time of Teilhard, the notion of telepathy was not yet ridiculed in scientific circles. His core message here is however his emphasis on the importance of *networks* (radio and television) for the formation of the Noosphere.

But I am also thinking of the insidious growth of those astonishing electronic computers which, pulsating with signals at the rate of hundreds of thousands a second, not only relieve our brains of tedious and exhausting work but, because they enhance the essential (and too little noted) factor of "speed of thought," are also paving the way for a revolution in the sphere of research.

Teilhard points out the other key ingredient of the future rise of the internet: *computers*. The sheer processing speed of computers is indeed what makes them so efficient compared to a human brain -especially once we learn how to program them and interface them with the world. In the same paragraph, Teilhard has emphasized the importance of both networks and computers, but has *not* linked them explicitly. Yet he is very close to formulating something like the birth of the Internet. In fact, Teilhard, though prescient, underestimates the full impact of computers. Now we know that their impact is not only on research, but on society as a whole.

And there are other forms of technical equipment, such as the electronic microscope whereby our sensory vision, the principal source of our ideas, has been enabled to leap the optical gap between the cell and the direct observation of large molecules. There is a school of philosophy which smiles disdainfully at these and kindred forms of progress. "Commercial machines," we hear them say, "machines for people in a hurry, designed to gain time and money." One is tempted to call them blind, since they fail to perceive that all these material instruments, ineluctably linked in their birth and development, are finally nothing less than the manifestation of a particular kind of super-Brain, capable of attaining mastery over some supersphere in the universe and in the realm of thought. "Everything for the individual!"-such was the reaffirmation of my brilliant friend, Gaylord Simpson, in a recent outburst [Note 11: George Gaylord Simpson, "The Role of the Individual in Evolution," *Journal of the Washington Academy of Sciences*, vol. 31, no. 1, 1941.] of antitotalitarian fervor.

The rise of technology is still not easy to digest for many because it transforms our societies and challenges our values at an accelerating pace. However, in contrast to some transhumanist visions, Teilhard’s vision seeks to integrate technology with human beings and evolution, and orient them all toward the awe-inspiring noosphere. Since he was a Christian, his visions (see especially Teilhard de Chardin 1959b) can also potentially help Christianity find ways to embrace rather than reject the transformations we are living through now.

But let us grasp this point clearly. No doubt it is true, scientifically speaking, that no distinct center of superhuman consciousness has yet appeared on earth (at least in the *living world*) for which it may be claimed or predicted that one day it will exercise a centralizing function, in relation to associated human thought, similar to the role of the individual "I" in relation to the cells of the brain.

Teilhard touches on the delicate and fascinating question of the noosphere’s identity, its self-consciousness or “I”. Consciousness may be a feature arising within any sufficiently complex information processing system like the brain, or the Internet. As Tononi and Edelman (1998) point out “neural processes associated with conscious experience are highly integrated and highly differentiated”. We see again this interplay between integration and differentiation, and sufficient integration and differentiation at the noosphere level may indeed lead to a new form of global self-consciousness. Another vision of a planetary consciousness is one of mindplexes composed of human and artificial minds (Montes and Goertzel 2019), which all have a “theater of consciousness” and interact tightly to create a new society of mind (Minsky 1986) at a planetary scale.

This discussion has also to do with centralized versus distributed architectures. The brain can be seen as a decentralized system, where the “I” emerges as a particular kind of activation and control in a global workspace (Baars 1988; Dehaene, Kerszberg, and Changeux 1998; Dehaene 2014). Yet the nervous system is also a very centralized system that controls bodily functions. Taking the global workspace as a model, it raises the question of what would be its analogue in the noosphere?

But that is far from saying that, influenced by the links which unite them, our grouped minds working together are not capable of achieving results which no one member of the group could achieve alone, and from which every individual within the collective process benefits "integrally," although still not in the total sense. We have only to consider any of the new concepts and intuitions which, particularly during the past century, have become or are in process of becoming the indestructible keystones and fabric of our thought -the idea of the atom, for example, or of organic Time or Evolution. It is surely obvious that no man on earth could alone have evolved them; no one man, thinking by himself, can encompass, master or exhaust them; yet every man on earth shares, in himself, in the universal heightening of consciousness promoted by the existence in our minds of these new concepts of matter and new dimensions of cosmic reality. It is not a question of simple repetitive "summation" but of synthesis. Not, it is true (at least not yet, here below) synthesis pushed to the point where it calls into being some new kind of autonomous supercenter in the depths of the synthesized, but a synthesis which at least suffices to erect, as though it were a vault above our heads, a sphere of mutually reinforced consciousness, the seat, support and instrument of supervision and superideas. No doubt everything proceeds from the individual and in the first instance depends on the individual; but it is on a higher level than the individual that everything achieves its fulfilment.

We have touched upon the apparatus of heredity, machinery and mind. It would be rash and often absurd to attempt to pursue further, and in detail, the comparison between the organism of the individual and that of the Noosphere. But the fact that the general line of analogy is valid and fruitful seems to me to be definitely proved by the very remarkable fact that these three systems, taken in conjunction, not only form a complementary and coherent whole, consistent within itself, but, which is even more easy of demonstration, that this whole is capable of breaking into motion and of working -that it *functions*, in a word.

Here again we see Teilhard’s non-reductionist mind at work, taking into account the three systems developing and acting *together* (heredity, machinery and mind). To understand in detail the mechanism of how they actually work in concert is of course a new challenge which may be at least as complicated for the noosphere as it is for a human body.

# 3. The Physiology of the Noosphere

ONE OF THE most impressive effects of the power of collective vision which is conferred upon us by the formation of a common brain is the perception of "great slow movements," so vast and slow that they are only observable over immense stretches of time. The currents that give birth to sidereal systems; the folds and upthrusts that form mountains and continents; the ebb and flow within the biosphere-in each case what we had supposed to be the extreme of immobility and stability is discovered to be a state of fundamental and irresistible movement.

That even the most apparently stable structures and objects (stars, mountains, animals, etc.) have had a long history is a major contribution of science, and contributes to major shifts in worldviews.

So it is with the Noosphere. I have already attempted a sort of anatomy of the major organs of the Noosphere. It remains for me to show that these separate parts, planetary in their dimensions, are not designed to remain in a state of rest. The formidable wheels turn, and in their combined action hidden forces are engendered which circulate throughout the gigantic system.

One could question that movement is really an outcome of the formation of the noosphere. That the noosphere moves may be an attractive idea given the overarching organic or animal analogy, because animals have evolved brains in order to move in their environment. However, it is not clear that the Earth would need the capacity for comparable movement through space in any foreseeable future. At most, the Earth might need to move to avoid a massive collision with an asteroid, or to escape the red giant phase of the Sun (Korycansky, Laughlin, and Adams 2001), but these scenarios are either unlikely or extremely far in the future. However, if “moving” means moving parts of the Earth itself, it is accurate that humans and machines are now moving more matter (sediments and rock) annually than all natural processes such as erosion and rivers (Arlind Boshnjaku 2012, 1:31).

What goes on around us in the human mass is not merely a flurry of disordered movement, as in a gas; something is purposefully stirring, as in a living being.

This is a straightforward teleological or directional claim. At first sight, the issue of seeing order or disorder looks like a subjective decision. And indeed, it has been argued that seeing the universe as random versus planful is a fundamental aspect of our psychological worldview (Koltko-Rivera 2004, 35). There have always been “collapsologists” emphasizing chaos and disorder, as opposed to optimistic and progressive thinkers seeing rising order. On this spectrum, Teilhard obviously emphasizes order. This optimistic attitude makes sense for any futurist, since it helps inspire and empower: as Kevin Kelly (2014) wrote “Over the long term, the future is decided by optimists.” An optimistic attitude requires courage, action, whereas the pessimist attitude can lead to resignation and inaction. Obviously, the two attitudes have pros and cons, and need to be in a constructive dialectical dialogue. The overoptimistic might miss threats and dangers, while the overpessimistic might miss opportunities.

Let us try to gain some understanding of this vast internal process of which we are all a part and to which we all contribute, almost without knowing it. At the heart of the entire movement, like the mainspring of a clock, there reappears, in identifiable form, what we have termed the inflexion of human stems upon themselves. It was of this mysteriously compelled in-folding, as I have said, that the human race was born.

The word “mysteriously” points to a non-scientific attitude, given that the goal of science is precisely to shed light on mysteries (e.g. Sagan 1995). Of course, there are many unknowns in science, but Teilhard may have chosen this language to make space for a religious interpretation of the birth of the human race.

I will now add that it is through the continued operation of the same movement that the race persists and functions. Indeed, we have only to open our eyes to be as it were spellbound by the dazzling vision, the spectacle of human shoots caught in the combined play of irresistible forces which slowly but surely continue to close and coil about us. Despite the havoc of war, the population on the limited surface of this planet which bears us is increasing in almost geometrical progression; while at the same time the scope of each human molecule, in terms of movement, information and influence, is becoming rapidly coextensive with the whole surface of the globe. A state of tightening compression, in short;

It is remarkable that Teilhard remains so positive just after World War II. He is correct about the geometrical progression of human population. It is also remarkable that he uses the word “compression”, a notion now adopted by futurists to describe the overall acceleration of technology (e.g. Smart 2009). In essence, Teilhard has identified both deep time (what Teilhard called “great slow movements”) and compressed time, two almost paradoxical temporalities affecting our orientation as we enter the Anthropocene (Shoshitaishvili 2020).

but, even more, thanks to the biological intermingling developed to its uttermost extent by the appearance of Reflection, a state of organized compenetration, in which each element is linked with every other. No one can deny that a network (a world network) of economic and psychic affiliations is being woven at ever increasing speed which envelops and constantly penetrates more deeply within each of us. With every day that passes, it becomes a little more impossible for us to act or think otherwise than collectively.

The expression “penetrates more deeply within each of us” might become literally true as interfaces between humans and machines become increasingly transparent, for example with neural implants connected to the internet (e.g. Heylighen 1999a; Musk 2019).

What is the significance of this multiform embrace, both external and internal, against which we struggle in vain? Can it mean that, caught in the ramifications of a sightless mechanism, we are destined to perish by stifling each other? No. For, as the coil grows tighter and the tension rises, the forces of super-compression in the vast generator find an effective outlet.

We begin to catch sight of it in the study of an all too familiar phenomenon, disquieting in appearance, but in fact highly revealing and reassuring – *the phenomenon of unemployment*. Owing to the extraordinarily rapid development of the machine, a rapidly increasing number of workers, running into tens of millions, are out of work. The experts gaze in dismay at this economic apparatus, their own creation, which, instead of absorbing all the units of human energy with which they furnish it, rejects an increasing number, as though the machine they devised were working to defeat their purpose. Economists are horrified by the growing number of idle hands.

Teilhard makes an argument similar to that of Keynes (1932) who saw the coming of technological unemployment, or the impactful book by David Rifkin (2014) *The zero marginal cost society*. Although he did not cite Teilhard, Rifkin (2014, 302) expressed Teilhardian conclusions, such as “Connecting every thing with every being -the Internet of Things- is a transformational event in human history, allowing our species to empathize and socialize as a single extended human family for the first time in history”.

Why do they not look a little more to biology for guidance and enlightenment? In its progress through a million centuries, mounting from the depths of the unconscious to consciousness, when has Life proceeded otherwise than by releasing psychic forces through the medium of the mechanisms it has devised? We have only to consider the evolution of the nervous system in the animal series, proceeding by chronological stages over a great period of time.

Here the evolution of nervous system is used as an inspiring analogy for apprehending the future of society. One can interpret the release of “psychic forces” as the emergence in evolution of new information markers and control, such as RNA, DNA, nervous systems, and culture (e.g. Turchin 1977; Dawkins 1995). The basic structure of the brain is useful to explore what could be the future development of the noosphere (or global brain) (de Rosnay 2012, 30–35; Vidal, Heylighen, and Weinbaum 2013). According to this modern brain analogy, the noosphere (or global brain) would go through three major stages: (1) a reptilian, connective phase, forming a kind of planetary nervous system (see also Helbing et al. 2012); (2) a limbic, collective dynamics eliciting emotions and stiring motivation, such as the current social networks; (3) the neocortex phase, dealing with abstractions, imagination, awareness, and unlocking the faculty of global imagination (e.g. scientific computer simulations), as well as the faculties of collective reasoning and decision making. The three are developing in parallel, but it is clear that the third phase is today still the least developed.

Or, let the theorists consider themselves. How are they capable of reasoning at all, if not because, within them, their visceral system has been taught to function automatically, while around them society is so well organized that they have both the strength and the leisure to calculate and reflect? What is true for each individual man is precisely what is happening at this moment on the higher level of mankind. Like a heavenly body that heats as it contracts, such, and in a twofold respect, is the Noosphere: first, in intensity, the degree in which its tension and psychic temperature are heightened by the coming together and mutual stimulation of thinking centers throughout its extent; and also quantitatively, through the growing number of people able to use their brains, because they are free from the need to labor with their hands. So that, to attempt to suppress unemployment by incorporating the unemployed in the machine would be against the purpose of Nature and a biological absurdity.

What Teilhard points is a transition from human work being primarily manual, manipulating matter-energy, to work on information and knowledge which makes use of brain power. At a societal level, it is only once the matter-energy needs have been automated that humans can afford to fully focus on informational work. The same holds at the individual level of the body. If you had to consciously think about making your balance, breathing and heartbeats function, there wouldn’t be any room, any freedom to do anything else. So unemployment is a side-effect of automation, but can also be seen as a sign that this automation is freeing human energy to do more sophisticated tasks.

The Noosphere can function only by releasing more and more spiritual energy with an ever higher potential.

Of course the notion of “spiritual energy” is problematic from a scientific standpoint to say the least. One secular interpretation it is that machines free the physical, matter-energy labor of humans, and therefore provide more opportunity for creativity and the expression of human mental or spiritual energy.

To all this, you may remark as follows: 'Very well; let us agree that the combined effect of phyletic intertwining and mechanical progress causes life to boil over. But, in that case, and surely it is the root of the matter, how are we to canalize and use the rising tide of liberated consciousness that is still so crude and unformed?' My answer is: 'By transforming it.' And, at this point, having invited you to reflect upon the phenomenon of unemployment, I will draw your attention to another and no less universal phenomenon, equally characteristic of the present age – *the phenomenon of research*.

Understanding, discovery, invention ... from the first awakening of his reflective consciousness, Man has been possessed by the demon of discovery; but, until a very recent epoch, this profound need remained latent, diffused, and unorganized in the human mass. In every past generation, true seekers, those by vocation or profession, are to be found; but, in the past, they were no more than a handful of individuals, generally isolated, and of a type that was virtually abnormal -- the 'inquisitive.' Today, without our having noticed it, the situation is entirely changed. In fields embracing every aspect of physical matter, life, and thought, the research workers are to be numbered in the hundreds of thousands, and they no longer work in isolation, but in teams endowed with penetrative powers that, it seems, nothing can withstand. In this respect, too, the movement is becoming generalized and is accelerating to the point where we must be blind not to see in it an essential trend in human affairs.

The growth of science is continuing as Teilhard notices. For example a UNESCO document (2021, 46) reports that there were 8.8 million full-time equivalent researchers worldwide in 2018, representing a growth of 13.67% since 2014. Valentin Turchin (1977) wrote *The Phenomenon of Science*, echoing Teilhard’s *The Phenomenon of Man*, and argues that the scientific control of scientific activity is the latest metasystem transition yet to take place.

Research which, until yesterday, was a luxury pursuit, is in the process of becoming a major, indeed the principal function of humanity.

Turchin (1977, 242) has shown that the growth of science is the most significant of current exponential trends: “Along with science other quantitative characteristics of the human race are growing exponentially: the total number of people and the total volume of production of material goods. But science significantly surpasses them in growth rate. The growth rates of population, production, and science are roughly in the ratio 1:2:4.”. The growth of researchers and publications is impressive, but I would not go so far as Teilhard to say that science becomes the principal function of humanity. Such an assertion may simply be expressing a professional bias of the scientist, as researchers represent “only” 0.11% of the human population even in 2018. Many human activities are still essential to make the superorganism work, as can be illustrated by Miller’s (1978) 20 living subsystems. From a systemic perspective, all subsystems need to be functional for the whole to function properly.

As to the significance of this great event, I, for my part, can see only one way to account for it. It is that the enormous surplus of free energy released by the in-folding of the Noosphere is destined, by a natural evolutionary process, to flow into the construction and functioning of what I have called its 'Brain.' As in the case of all the organisms preceding it, but on an immense scale, humanity is in the process of 'cerebralizing' itself.

Teilhard employs a hydraulics analogy when he writes of the “enormous surplus of free energy released by the infolding of the Noosphere”. Imagine a fixed amount of flow that can go through evolution on Earth: the in-folding creates pressure on existing earth systems, which is released in the construction of the Noosphere. This is a typical way of thinking for an orthogenesist. The two manifestations of this free energy that he describes are the phenomenon of unemployment and the phenomenon of research.

And our proper biological course, in making use of what we call our leisure, is to devote it to a new kind of work on a higher plane: that is to say, to a general and concerted effort of vision. The Noosphere, in short, is a stupendous thinking machine.

Teilhard emphasizes that new kinds of possible activities are emerging, that were considered unessential leisure before, but that are essential at a noospheric level.

It is in this sense alone, as I believe, that the horizon appears and we can gain a clear view of the human world surrounding us. In harmony with the cosmic impulse which leads to the constant disintegration of atoms and the attendant release of energy, Life (though probably localized on a few rare planets) compels us increasingly to view it as an underlying current, in the flow of which matter tends to order itself upon itself, with the emergence of consciousness. On the one hand, we have physical radiation bound up with disintegration; and, on the other hand, psychic radiation bound up with an ordered aggregation of the stuff of the universe.

Teilhard, probably influenced by Bergson (1984), articulates the two fundamental trends of the universe which contrast each other: in modern terms, there are the second law of thermodynamics, and the growth of complexity. Today, complexity sciences have started to explain much more about the ordering of the universe through the interaction of these two fundamental trends, and one need not to resort to a vague notion such as “psychic radiation”.

In the eyes of nineteenth century science, the interiorization of the world, leading to the phenomenon of Reflection, might still pass for an accident and an anomaly.

Unfortunately, this science-based vision of humanity as a cosmic accident has been and still is enduring (e.g. Weinberg 1993).

We now see it to be a clearly defined process, coextensive with the whole of reality. Complexification due to the growth of consciousness, or consciousness the outcome of complexity: experimentally, the two terms are inseparable. Like a pair of related quantities, they vary simultaneously.

The word “experimentally” seems inappropriate to me here, as there are no scientific experiments to support such a general statement. Also, even granting that the growth of consciousness and of complexity go together, this formulation risks simply conflating the two concepts. We’ll further discuss the law of complexity-consciousness below.

And surely, it is within this generalized cosmic process that the Noosphere, a particular and extreme case, has its natural place and takes its shape: the maximum of complication, represented by phyletic in-folding, and in consequence the maximum of consciousness, emerging from the system of individual brains, coordinated and mutually supporting. And, this is exactly what was to be expected.

But, it is assuredly a remarkable coincidence that, in justifying the organic interpretation of the Phenomenon of Man, as we have sought to do, we should also be paving the way for a reasonable forecast as to our future destiny, and the fate which is reserved for us at the end of Time.

I do agree that the noosphere stage of evolution is a fundamental level on which further speculations about the future should build.

# 4. The Phases and Future of the Noosphere

We have found it possible to express the social totalization which we are undergoing in terms of a clearly identifiable biological process. Proceeding from this, we may surely look into the future and predict the course of the trajectory we are describing. Once we have accepted that the formation of a collective human organism, a Noosphere, conforms to the general law of recurrence, which leads to the heightening of Consciousness in the universe as a function of complexity, a vast prospect opens before us.

Teilhard is more assertive here, casting the current stage of “social totalization” as “a clearly identifiable biological process” and hence making the biological analogy explicit and central. This move needs to be contrasted with the beginning of the article where Teilhard stresses the delicacy of using an organic analogy: “How are we to escape from metaphor without falling into the trap of establishing absurd and oversimplified parallels which would make of the human species no more than a kind of composite, living animal?”. Interpreted too simplistically, the superorganism analogy can easily be abused to justify the worst totalitarian regimes. As a matter of fact, fascism, nazism and stalinism have all abused the (super)organism language for their own agenda (see respectively Gini 1927; Hitler 1939; Ree 1993). Here, Teilhard seems to have let down his guards with the analogy, and one should be wary with the word “social totalization” -and later in the conclusion he’ll use the words “center of power” and “totalitarian mechanism”. Unfortunately, this negligence and drift are not isolated cases, as recently John P. Slattery (2017) raised even redder flags by claiming that Teilhard “unequivocally supported racist eugenic practices, praised the possibilities of the Nazi experiments, and looked down upon those who he deemed “imperfect” humans.” This obviously stirred some debate, and Slattery wrote a shorter blog article (Slattery 2018a), which received criticisms by Teilhard experts (Canzona 2018; Haught 2019), and to which Slattery replied (respectively in Slattery 2018b; and 2019).

To what regions and through what phases may we suppose that the extension of the rising curve of hominization will carry us?

Immediately confronting us (indeed, already in progress) we have what may be called a 'phase of planetization.' It can truly be said, no doubt, that the human group succeeded long ago in covering the face of the earth and that, over a long period, its state of zoological ubiquity has tended to be transformed into an organized aggregate. But, it must be clear that the transformation is only now reaching its point of full maturity. Let us glance over the main stages of this long history of aggregation. First, in the depths of the past, we find a thin scattering of hunting groups spread here and there throughout the Ancient World. At a later stage, some fifteen thousand years ago, we see a second scattering, very much more dense and clearly defined: that of agricultural groups installed in fertile valleys -- centers of social life where man, arrived at a state of stability, achieved the expansive powers which were to enable him to invade the New World. Then, only seven or eight thousand years ago, there came the first civilizations, each covering a large part of a continent. These were succeeded by the real empires. And so on ... patches of humanity growing steadily larger, overlapping, often absorbing one another, thereafter to break apart and again reform in still larger patches.

What Teilhard outlines here has now grown into the academic field of Big History (e.g. Spier 1996; Christian 2004), though he emphasizes in this passage human over cosmic evolution, while big history tends to connect both. Note that Teilhard weaves this broader connection in *The Phenomenon of Man.*

As we view this process – the spreading, thickening, and irresistible coalescence – can we fail to perceive its eventual outcome? The last blank spaces have vanished from the map of mankind. There is contact everywhere, and how close it has become! Today, embedded in the economic and psychic network which I have described, two great human blocks alone remain confronting one another. Is it not inevitable that, in one way or another, these two will eventually coalesce?

Teilhard probably implicitly refers to the US, the Soviet Union and the Cold War.

Preceded by a tremor, a wave of 'shared impulse' extending to the very depth of the social and ethnic masses, in their need and claim to participate, without distinction of class or color, in the onward march of human affairs, the final act is already visibly preparing.

This inspiring prose points to a real phenomenon that is arising today, namely global compassion (see e.g. Ekman 2014). This is not just wishful thinking, but has been facilitated by efficient telecommunication networks, which allow us to empathize with victims and react against injustices happening nearly anywhere in the world. This form of interconnection has been a key contribution to the global decrease in violence (Pinker 2011).

Although the form is not yet discernible, mankind tomorrow will awaken to a 'pan-organized' world.

The word “pan-organized” is a neologism, but may be interpreted as referring to a kind of global governance, where every smaller entity (nation, society) will be integrated into a larger world organization.

But, and we must make no mistake about this, there will be an essential difference, a difference of order, between the unitary state towards which we are moving, and everything we have hitherto known. The greatest empires in history have never covered more than fragments of the earth. What will be the specifically new manifestations which we have to look for in the transition to totality? Until now, we have never seen mind manifest itself on this planet, except in separated groups and in the static state. What sort of current will be generated, what unknown territory will be opened up, when the circuit is suddenly completed?

These are excellent questions, and there is still much debate on the future of governance. The United Nations, or the World Health Organization are early examples of intergovernmental organizations aiming at global governance, but their scope and power is limited in comparison to what would be needed for an effective global governance that would be able to successfully regulate the power of whole nations.

I believe that what is now being shaped in the bosom of planetized humanity, is essentially a rebounding of evolution upon itself. We all know about the real or imaginary projectiles whose thrust is renewed by the firing of a series of staged rockets. Some such procedure, it seems to me, is what Life is preparing at this moment, to accomplish the supreme, ultimate leap.

One could very much question the idea that this transition would be the “supreme, ultimate leap”. It may certainly seem to be from our current perspective, since our limited human cognition struggles to apprehend a planetary transition. However, many major transitions have happened in past cosmic evolution, and arguably, many are to come in the future. Various thinkers have offered speculations on further reaching ultimate, universal futures (see e.g. Tipler 1995; Deutsch 1997; Smart 2012; Vidal 2014b).

The first stage was the elaboration of lower organisms, up to and including Man, by the use and irrational combination of elementary sources of energy received or released by the planet. The second stage is the super-evolution of man, individually and collectively, by the use of refined forms of energy scientifically harnessed and applied in the bosom of the Noosphere, thanks to the coordinated efforts of all men working reflectively and unanimously upon themselves.

Teilhard touches upon a central theme in modern Big History and energetics: the methods of harnessing energy both in living beings and in societies (see e.g. Niele 2005).

Who can say whither, coiled back upon our own organism, our combined knowledge of the atom, of hormones, of the cell, and the laws of heredity will take us? Who can say what forces may be released, what radiations, what new arrangements never hitherto attempted by Nature, what formidable powers we may henceforth be able to use, for the first time in the history of the world?

Teilhard asks provocative questions here, typical of modern transhumanist discussions.

This is Life setting out upon a second adventure from the springboard it established when it created humankind.

This statement is an important insight and reflects Teilhard’s understanding that the noosphere marks a major evolutionary transition. Harvard roboticist Hans Moravec (1988, 3–4) has also argued that what is happening with the rise of machines is comparable only to the origin of life itself. Recently, Kevin Kelly (2010) argued that technology has a drive of its own.

But all this is no more than the outward face of the phenomenon. In becoming planetized, humanity is acquiring new physical powers which will enable it to super-organize matter.

The expression “super-organize” is of course open to many interpretations. One can note that the exponential trends in technological progress are largely driven by material innovations on ever smaller scales (Magee 2012), yielding a finer and finer control of matter. This trend of civilizational development is called the “Barrow scale” (Barrow 1998; Vidal 2014b; Smart 2012), as a complement to the trend of increased energy use captured by the “Kardashev scale” (Kardashev 1964).

And, even more important, is it not possible that, by the direct converging of its members, it will be able, as though by resonance, to release psychic powers whose existence is still unsuspected? I have already spoken of the recent emergence of certain new faculties in our minds, the sense of genetic duration and the sense of collectivity. Inevitably, as a natural consequence, this awakening must enhance in us, from all sides, a generalized sense of the organic, through which the entire complex of inter-human and inter-cosmic relations will become charged with an immediacy, an intimacy, and a realism such as has long been dreamed of and apprehended by certain spirits particularly endowed with the 'sense of the universal,' but which has never yet been collectively applied.

One could interpret the internet and the web as a mechanism to release the “psychic powers”. Teilhard anticipates the rise of ecological awareness here, what he calls a “generalized sense of the organic”. Although the real challenge for the future is not only ecology, but the integration in a synergistic way of ecology and human societies (Odum 2001). Regarding the entire complex of “inter-cosmic relations”, Teilhard actually wrote “*intra*-cosmic relations” in the French original. It obviously makes more sense because “inter-cosmic relations” would imply that there is more than one cosmos. It may be too early to establish such a deep connection with the cosmos, comparable to the deep connection human beings may achieve with the Earth. Although it may be argued that this is the next logical step to consolidate after planetary awareness and integration. This has indeed been explored by many cosmic scientists and philosophers (see e.g. Sagan 1973; Swimme and Berry 1992; Goertzel 2010; Stewart 2010; Young 2012; Lem 2013; Vidal 2014b).

And it is in the depths and by grace of this new inward sphere, the attribute of planetized Life, that an event seems possible which has hitherto been incapable of realization: I mean the pervasion of the human mass by sympathy, a communication of mind and spirit that will make the phenomenon of telepathy, still sporadic and haphazard, both general and normal. But, above all, it will be a state of active sympathy, in which each separate human element, breaking out of its insulated state under the impulse of the high tensions generated in the Noosphere, will emerge into a field of prodigious affinities, which we may already conjecture in theory.

One could note that the phenomenon of telepathy has indeed become general and normal... but through the technological solution of the internet! The noosphere and other global- or super- brains emphasize the mental, thinking side. Implicitly relying on the organismic analogy, the question of a “planetary heart” or “global heart” is no less fascinating to ponder. As we mentioned earlier, a central indication of the rise of such a global heart is the global decline of violence (Pinker 2011).

For, if the power of attraction between simple atoms is so great, what may we not expect if similar bonds are contracted between human molecules?

Teilhard’s metaphor here is the transition from physics to chemistry. He implicitly refers to the fact that atoms bonding into molecules create a much larger variety of possible combination and complexity than the limited number of atoms of the periodic table of elements. Similarly, connecting humans (and machines) on a global scale opens up a formidable combinatory space.

Humanity, as I have said, is building its composite brain beneath our eyes. May it not be that tomorrow, through the logical and biological deepening of the movement drawing it together, it will find *its heart*, without which the ultimate wholeness of its powers of unification can never be fully achieved? To put it in other words, must not the constructive developments now taking place within the Noosphere, in the realm of sight and reason, necessarily also penetrate to the sphere of feeling?

Again, the question of what this global sphere of feeling could be is not clear yet, but is well worth exploring and keeping in mind (and heart).

The idea may seem fantastic when one looks at our present world, still dominated by the forces of hatred and repulsion. But, is not this simply because we refuse to heed the admonitions of science, which is daily proving to us, in every field, that seemingly impossible changes become easy and even inevitable, as soon as there is a change in the order of the dimensions?

I am not sure what Teilhard means with “a change in the order of the dimensions”, possibly he refers to the idea that a quantitative increase can lead to a qualitative change. For example consciousness might emerge only with hundreds of billions of neurons. A similar argument has been made by global brain pioneer Peter Russell (1995), arguing that as human population reaches 10 billions, a transition would occur. As a note, the population growth of technological artefacts is much more rapid than the population of humans, which is an argument to say that the transition would likely involve machines centrally.

To me, two things, at least, now seem certain. The first is that, following the state of collective organization we have already achieved, the process of planetization can only advance ever further in the direction of growing unanimity.

We can definitely grant that Teilhard was correct here, since the world has evolved into a much more globalized entity than it was in 1947.

And, the second is that this growth of unanimity, being of its nature convergent, cannot continue indefinitely without reaching the natural limit of its course. Every cone has an apex.

Here Teilhard refers to a geometrical metaphor, the cone. I personally find it less inspiring than other biological metaphors, in part because geometrical objects are so much simpler than biological systems. I also do not believe that the noosphere is the end of evolution.

In the case of this human aggregation, how shall we seek, not to imagine, but to define the supreme point of coalescence? In terms of the strictly phenomenal viewpoint which I have adopted throughout this paper, it seems to me that the following may be said:

What, at the very beginning, made the first man was, as we know, the heightening of the individual consciousness to the point where it acquired the power of Reflection. And, the measure of human progress during the centuries which followed is, as I have sought to show, the increase of this reflective power through the interaction, or conjugated thought, of conscious minds working upon one another. Well, what will finally crown and limit collective humanity at the ultimate stage of its evolution, is and must be, by reason of continuity and homogeneity, the establishment of a focal point at the heart of the reflective apparatus as a whole.

If we concede this, the whole of human history appears as a progress between two critical points: from the lowest point of elementary consciousness, to the ultimate noospherical point of Reflection. In biological terms, humanity will have completed itself and fully achieved its internal equilibrium only when it is psychically centered upon itself (which may yet take several million years).

Here Teilhard gives us a time horizon for the ultimate phase of the noosphere (several million years). Of course, such a prediction is impossible to test given our lifetimes, but it is interesting that even this timescale is *short* for the paleobiologist that he is, manipulating dozen, hundreds or thousands of millions of years.

What the stage of being “psychically centered upon itself” may mean, or how to know when it has arrived is also open to interpretation. One interpretation is to say it is precisely planet earth’s reflection about itself, a global consciousness. Another speculative but more technical interpretation is to imagine a massively distributed network of humans and AI, reading inputs from the billions of sensors deployed by the internet of things and giving rise to an emergent planetary reflection, or consciousness.

In a final effort of thought, let us remove ourselves to that ultimate summit where, in the remote future, but seen from the present, the tide which bears us reaches its culmination. Is there anything further to be discerned beyond that last peak etched against the horizon? Yes and no.

Teilhard makes one final speculative effort to attempt to see beyond the noosphere.

In the first place, no, because, at that mysterious pole crowning our ascent, the compass that has guided us runs amok. It was by the law of 'consciousness and complexity' that we set our course: a consciousness becoming ever more centered, emerging from the heart of an increasingly vast system of more numerous and better organized elements. But, now we are faced with an entirely new situation: for the first time, we have no multiple material under our hands. Unless, as seems infinitely improbable, we are destined by contact with other thinking planets, across the abysses of space and time, some day to become integrated within an organized complex composed of a number of Noospheres, humanity, having reached maturity, will remain alone, face to face with itself. And, at the same time, our law of recurrence, based on the play of interrelated syntheses, will have ceased to operate.

The “law of ‘consciousness and complexity’” is a central theme in Teilhard’s thinking. Of course, introducing a new scientific “law” is no small claim, and Teilhard may be accused of not providing a precise enough formulation and justification. Let us try to support it with some insights from cybernetics and evolution. Mind, cognition, intelligence and consciousness are linked and must grow to deal with an environment that becomes more rich and complex. More precisely, this comes from Ashby's *law of requisite variety* and the *law of requisite knowledge* (Ashby 1956; Heylighen and Joslyn 2001): as the environment becomes more complex, organisms need to become better in making sense of their situation, i.e. become more intelligent, knowledgeable and conscious of what is going on, and must have the requisite knowledge in order to perform the right actions. If they do not, they will be unable to solve their problems, which means that they will disappear through a process of natural selection. Also, another fundamental cybernetic insight is the *good regulator theorem* stating that every good regulator of a system must be a model of that system (Conant and Ashby 1970). Therefore, since regulation is essential to survival in the long term, organisms must make models of their environments. And since the environment grows more and more complex, the models must also become so, which requires more sophisticated and capable forms of consciousness able to create them.

Teilhard is correct to point out that further complexity-consciousness is unlikely on universal spatial scales, mostly because of the vast distances separating stars, planets and thus putative other noospheres. High complexity, requires compression, short distances, to increase the number of interactions, computations, and feedback loops, and thus learning. Even staying on Earth, we are actually very far from the ultimate limits of computation, of controlling matter with the smallest possible information markers (Landauer 1961; Lloyd 2000). Nevertheless, I would not exclude a very slow communication or interaction between extraterrestrial civilizations in the galaxy, even if it may never develop into a “galactic mind”.

So, in one sense, it all seems to be over; as though, having reached its final point of Noospheric Reflexion, the cosmic impulse towards consciousness has become exhausted, condemned to sink back into the state of disintegration implacably imposed on it by the laws of stellar physics. But, in another sense, nothing will be ended. For, at this point, and at the height of its powers, something else comes into operation, a primary attribute of Reflection concerning which we have hitherto said nothing – the will to survive. In reflecting upon itself, the individual consciousness acquires the formidable property of foreseeing the future, that is to say, death.

Teilhard’s views about the far-future of intelligent life in the universe are compatible with modern cosmological extrapolations (e.g. Vidal 2014b, pt. 3). Indeed, it is the predictable death of our Sun that would motivate us either to migrate to another star, or to attempt stellar engineering. And the ultimate death represented by the heat death of the universe would motivate any intelligence in this universe to try to overcome it and reach one form or another of immortality (Vidal 2014a). The form that such “advanced” noospheres would take would follow the history of energy extraction innovation all the way to becoming star-eating civilizations or *stellivores*, whose technosignatures may be detectable within existing astrophysical data (Vidal 2014b, chap. 9; 2016; 2020).

And, at the same time, it knows that it is psychologically impossible for it to continue to work in pursuance of the purposes of Life unless something, the best of the work, is preserved from total destruction. In this resides the whole problem of action. We have not yet taken sufficient account of the fact that this demand for the Absolute, not always easily discernible in the isolated human unit, is one of the impulses which grow and are intensified in the Noosphere. Applied to the individual, the idea of total extinction may not, at first sight, appall us; but, extended to humanity as a whole, it revolts and sickens us. The fact is that, the more Humanity becomes aware of its duration, its number, and its potentialities – and also of the enormous burden it must bear in order to survive -- the more does it realize that, if all this labor is to end in nothing, then we have been cheated and can only rebel.

Planetary death does indeed seem intolerable, and a will towards immortality has been a great driver of human civilization (Cave 2012). Great minds such as Darwin (1887, 70) made similar comments regarding the unacceptability of the heat death of the universe.

In a planetized Humanity, the insistence upon irreversibility becomes a specific requisite of action; and it can only grow and continue to grow as Life reveals itself as being ever more rich, an ever heavier load. So that, paradoxically, it is at that ultimate point of centration which renders it cosmically unique, that is to say, apparently incapable of any further synthesis, that the Noosphere will have become charged to the fullest extent with psychic energies to impel it forward in yet another advance ...

In essence, Teilhard is correct that it is nearly impossible for us to foresee what the next stages of evolution will be. A (loose) analogy would be a cell that might be able to imagine what it is like to be part of a multicellular organism, but would not be able to imagine what a group of multicellular organisms could be, and have even less any idea of the scale and kind of organization that these new evolutionary complexes would consist of.

And, what can this mean, except that, like those planetary orbits which seem to traverse our solar system without remaining within it, the curve of consciousness, pursuing its course of growing complexity, will break through the material framework of Time and Space to escape somewhere towards an ultra-center of unification and consistence, where there will finally be assembled, and in detail, everything that is irreplaceable and incommunicable in the world.

And, it is here, an inevitable intrusion in terms of biology, and in its proper place in terms of science, that we come to the problem of God.

This may be the most obscure and yet most fascinating passage of this essay. First, he uses an astrophysical analogy of perhaps comets? It is an inertial analogy, proposing that the curve of consciousness has an impetus towards growth in complexity. Then he claims that this growth will break the frameworks of space-time. One could interpret it in a rather extreme futuristic way, arguing that a black hole is really the ultimate configuration of an extremely advanced civilization in the universe (Vidal 2011; Smart 2012). A more likely interpretation is a theological one, as God is indeed eternal, which means outside space-time, and the expression “irreplaceable and incommunicable” inspires feelings of mystery. But the real twist to traditional Christianity is that the “problem of God” appears when thinking about the far future! This statement is similar to the radical form of transhumanism defended by Ray Kurzweil, who ends his documentary Transcendent Man (Ptolemy 2011) with the provocative conditional: "if I was asked if God exists, I would say not yet". Although Teilhard’s evolutionary cosmology has indeed been interpreted in the light of modern transhumanism (Steinhart 2008), theologically, this passage can be better understood by noticing that Teilhard held a Scotish or Franciscan conception of incarnation -as opposed to the more common Thomist conception. This Scottish/Franciscan conception holds an organic view of the Christ, as the achievement of supernatural and *natural* order. Based on this, Teilhard argues that cosmic evolution is an ascent towards the Christ: cosmogenesis leads first to biogenesis, then noogenesis and is ultimately achieved in christogenesis, i.e. the omega point (see Teilhard de Chardin 1945, 210; Wildiers 1960, 92).

# Conclusion: The Rise of Freedom

Let us turn to cast an eye over the road that we have followed.

At the beginning, we seemed to see around us nothing but a disconnected and disordered humanity: the crowd, the mass, in which, it may be, we saw only brutality and ugliness. I have tried, fortified by the most generally accepted and solid conclusions of science, to take the reader above this scene of turmoil; and, as we have risen higher, so has the prospect acquired a more ordered shape. Like the petals of a gigantic lotus at the end of the day, we have seen human petals of planetary dimensions slowly closing in upon themselves.

Teilhard uses a beautiful botanical metaphor to speak about planetary convergence.

And, at the heart of this huge calyx, beneath the pressure of its infolding, a center of power has been revealed, where spiritual energy, gradually released by a vast totalitarian mechanism, then concentrated by heredity within a sort of super-brain, has, little by little, been transformed into a common vision growing ever more intense.

As with the earlier expression “social totalization”, “center of power” and “totalitarian mechanism” are highly problematic given their authoritarian associations. With a generous reading, one can note that the word “totalitarian” is not necessarily political, but can simply mean “in totality”, a nuance that exists in French but maybe less in English. Of course, this passage sums up his thinking that the further concentration of humans will be by -cultural- heredity towards a super-brain (noosphere).

In this spectacle of tranquillity and intensity, where the anomalies of detail, so disconcerting on our individual scale, vanish to give place to a vast, serene, and irresistible movement from the heart, everything is contained and everything harmonized in accord with the rest of the universe.

The oxymoron of tranquility coupled with intensity is not explained here. He probably means that an intense change can occur without major wars or destructive revolution. The movement from the heart could be read as both the decline of violence and the evolution of collaboration on larger and larger scales (see e.g. Stewart 2000).

Life and consciousness are no longer chance anomalies in Nature; rather, we find in biology a complement to the physics of matter. On the one hand, I repeat, the stuff of the world dispersing through the radiation of its elemental energy; and, on the other hand, the same stuff re-converging through the radiation of thought.

Today we could reframe this summary as the struggle between the second law of thermodynamics, what Teilhard calls “the stuff of the world dispersing through the radiation of its elemental energy” and the growth of complexity, or “re-converging through the radiation of thought”. Of course, the two radiations are of different nature: one is made of energy, and the other is about information. It is worth mentioning here that this “radiation of thought” can be totally naturalized today, which perhaps was not obvious to Teilhard, who was still influenced by Bergson’s (1984) commitment to vitalism.

The fantastic at either end: but, surely, the one is necessary to balance the other? Thus, harmony is achieved in the ultimate perspective and, furthermore, a program for the future: for, if this view is accepted, we see a splendid goal before us, and a clear line of progress. Coherence and fecundity, the two criteria of truth.

Teilhard’s optimism and future vision is remarkable here. The goal of collectively building the noosphere is indeed one that has the potential to unite humanity, in a positive way (complementing motivations to avoid negative paths, such as those leading toward climate change, nuclear war and other existential threats).

Is this all illusion, or is it reality?

It is for the reader to decide. But, to those who hesitate, or who refuse to commit themselves, I would say: 'Have you anything else, anything better to suggest, that will account scientifically for the phenomenon of man considered as a whole, in the light of his past development and present progress?'

Here Teilhard abuses logic and rhetoric. First, he puts forth a *bogus dilemma* (Pirie 2006, 24) with the question “is this all illusion, or is it reality?” Of course, Teilhard could be correct only partially, so the framing does not need to be either one or the other. Moreover, when he challenges anyone to come with alternative scenarios for the future, he is actually *shifting the burden of proof* (Pirie 2006, 149). Even if no better alternative were found, this would not be a convincing formal proof, as *damning the alternatives* (Pirie 2006, 44) does not necessarily lead to the right conclusion since, logically, all options might turn out to be wrong. Nevertheless, if one accept this forced choice, the way Teilhard argues is valid from a philosophical methodological point of view, as: “a position that, in comparison with its alternatives, encounters fewer and lesser difficulties than they do thereby deserves to be adopted - at least provisionally, until something better comes along” (Rescher 2006, 7).

Beyond these contestable argumentative tactics, however, it would be interesting to research if there were specific alternatives proposed at the time of publication. Nonetheless I largely agree that once we take the constraints of the mechanisms of past evolution and the observation of current global trends, the space of possibilities greatly narrows, and Teilhard’s vision has already turned out to be mostly correct.

You may reply to me that this is all very well, but is there not something lacking, an essential element, in this system which I claim to be so coherent? Within that grandiose machine-in-motion which I visualize, what becomes of that pearl beyond price, our personal being? What remains of our freedom of choice and action?

Teilhard addresses here the age-old philosophical question of freedom and free will, in the new context of the noosphere.

But, do you not see that, from the standpoint I have adopted, it appears everywhere – and is everywhere heightened? I know very well that, by a kind of innate obsession, we cannot rid ourselves of the idea that we become most masters of ourselves by being as isolated as possible. But, is not this the reverse of the truth? We must not forget that, in each of us, by our very nature, everything is in an elemental state, including our freedom of action. We can only achieve a wider degree of freedom by joining and associating with others in an appropriate way.

Complex systems are comprised of parts that are both autonomous, and interdependent. For example, cells have all the features of an autonomous living system (Miller 1978), and yet in a multicellular organism, they depend on the activities of other cells and other systems. On Earth, both unicellular and multicellular organisms co-exist, which shows that the transition to multicellularity is not inevitable for every living thing. Also, if you choose to live and manage to survive outside of society, you will indeed be freed from societal constraints, but there is no doubt that the range of possible actions is much greater for those who integrate into society (even if integration involves new constraints). This point is clear in our globalized world, consider the freedom to order almost any product via the internet or travel almost anywhere on Earth. These possibilities are obviously closed if you are isolated from society. Teilhard is right to point out that the degrees of freedom increase when we are connected to others.

Again, in complex systems there is a dynamic of both differentiation, “autonomization”, as well as integration (Heylighen 1999b). The autonomy and freedom of the parts is also a necessary condition for the robustness of complex organizations (see e.g. Beer 1972).

This is, to be sure, a dangerous operation since, whether it be a case of disorderly intermingling, or of some simple form of coordination, like the meshing of gear wheels, our activities tend to cancel one another out, or to become mechanical. We find this only too often in practice. Yet, it is also salutary, since the approach of spirit to spirit in a common vision, or shared passion, undoubtedly enriches all: in the case of a team, for example, or of two lovers. Achieved with sympathy, union does not restrict, but exalts the possibilities of our being.

In the simple examples he gives of gear wheels, there is indeed no autonomy or freedom of the parts, given the rigid constraints. The examples of two lovers or a team are excellent indeed, because ideally the *individual autonomy* could be maintained at least at times, and it would seem this autonomy underlies the “sympathy” Teilhard mentions, even while the joining together opens new emergent possibilities. This discussion is also central in the concept of a metasystem transition, where a new level of control emerges, which unlocks new possibilities, even if the process has to add new constrains to the lower-level systems. As Valentin Turchin (1995, 59) explains, this is a dangerous and delicate operation:

“The creative freedom of individuals is the fundamental engine of evolution in the era of Reason. If it is suppressed by integration, as in totalitarianism, we shall find ourselves again in an evolutionary dead end. This contradiction is real, but not unsolvable. After all, the same contradiction has been successfully solved on other levels of organization in the process of evolution. When cells integrate into multicellular organisms, they continue to perform their biological functions -metabolism and fission. The new quality, the life of the organism, does not appear despite the biological functions of the individual cells but because of them and through them. The creative act of free will is the 'biological function' of the human being. In an integrated super-being it must be preserved as an inviolable foundation, and the new qualities must appear through it and because of it. Thus the fundamental challenge that humanity faces is to achieve an organic synthesis of integration and freedom.”

We see this everywhere and every day, on a limited scale. Why should it not be worth correspondingly more on a vast and all embracing scale, if the law applies to the very structure of things? It is simply a question of tension within the field that polarizes and attracts. In the case of a blind aggregation, of some form of purely mechanical arrangement, the effect of large numbers is to materialize our activities. That is true. But, where it is a matter of unanimity realized from within, the effect is to personalize them and, I will add, to make them unerring. A single freedom, taken in isolation, is weak and uncertain and may easily lose itself in mere groping. But, a totality of freedom, freely operating, will always end by finding its road. And this, incidentally, is why, throughout this paper, without seeking to minimize the uncertainties inherent in Man's freedom of choice in relation to the world, I have been able implicitly to maintain that we are moving, both freely and ineluctably, in the direction of concentration by way of planetization.

A way to illustrate Teilhard’s point is that one can predict a coarse-grained future, which would not eliminate our local freedom, creativity, and surprise in the world. For example, we can predict that our children will find a lover and have children. Yet the triviality of this statement leaves a lot of freedom. How, when, or with whom our children will fall in love and what their children will be like remains full of surprises.

One might put it that determinism appears at either end of the process of cosmic evolution, but in antithetically opposed forms: at the lower end, it is forced along the line of the most probable for lack of freedom; at the upper end, it is an ascent into the improbable through the triumph of freedom. We may be reassured. The vast industrial and social system by which we are enveloped does not threaten to crush us, neither does it seek to rob us of our soul. The energy emanating from it is free, not only in the sense that it represents forces that can be used; it is moreover free because, in the whole, no less than in the least of its elements, it arises in a state that is ever more spiritualized.

A thinker such as Cournot [Note 12: Cournot, A.-A. *Considerations sur la Marche des idées et des Evenements dans les Temps modernes*. (Réédition Mentre. Vol. II, p. 178).] might still be able to suppose that the socialized group degrades itself biologically in terms of the individuals which comprise it. Only by reaching to the heart of the Noosphere (we see it more clearly today) can we hope, and indeed be sure of finding, all of us together and each of us separately, the fullness of our humanity.

What Cournot might have identified are examples of “bad” integration, where there is a degradation of the individuals and their freedom. A successful integration may hinge on the development of the noosphere’s “heart”, alongside a growing understanding of the evolutionary mechanisms leading to higher cooperation (Wilson 2019).

**Acknowledgments:** I thank Terrence Deacon, Francis Heylighen, Ben Kacyra, Boris Shoshitaishvili for insightful comments and corrections. This research received funding from the Kacyra Family Foundation (KFF) as part of its Human Energy Project (HEP).

# **References**

Arlind Boshnjaku. 2012. *Welcome to the Anthropocene*. <https://www.youtube.com/watch?time_continue=197&v=fvgG-pxlobk>.

Ashby, W. R. 1956. *An Introduction to Cybernetics*. New York: J. Wiley. <http://pespmc1.vub.ac.be/books/IntroCyb.pdf>.

Aunger, Robert, ed. 2000. *Darwinizing Culture*. Oxford University Press.

———. 2007a. “A Rigorous Periodization of ‘big’ History.” *Technological Forecasting and Social Change* 74 (8): 1164–78. doi:10.1016/j.techfore.2007.01.007.

———. 2007b. “Major Transitions in ‘big’ History.” *Technological Forecasting and Social Change* 74 (8): 1137–63. doi:10.1016/j.techfore.2007.01.006.

———. 2017. “Moral Action as Cheater Suppression in Human Superorganisms.” *Frontiers in Sociology* 2. Frontiers. doi:10.3389/fsoc.2017.00002. <https://www.frontiersin.org/articles/10.3389/fsoc.2017.00002/full>.

Baars, Bernard J. 1988. *A Cognitive Theory of Consciousness*. Cambridge [England] ; New York: Cambridge University Press.

Barberis, Daniela S. 2003. “In Search of an Object: Organicist Sociology and the Reality of Society in Fin-de-Siècle France.” *History of the Human Sciences* 16 (3). Sage Publications: 51–72.

Barrow, J. D. 1998. *Impossibility: The Limits of Science and the Science of Limits*. Oxford University Press, USA.

Barrow, J. D., and F. J. Tipler. 1986. *The Anthropic Cosmological Principle*. Oxford University Press.

Beer, Stafford. 1972. *Brain of the Firm: A Development in Management Cybernetics*. New York: Herder and Herder.

Bergson, Henri. 1984. *Creative Evolution*. University Press of America.

Berners-Lee, T. 1999. *Weaving the Web*. HarperBusiness New York.

Boehm, Christopher. 2012. *Moral Origins: The Evolution of Virtue, Altruism, and Shame*. New York: Basic Books.

Boulding, K. E. 1956. “General Systems Theory-The Skeleton of Science.” *Management Science* 2 (3): 197–208. <http://pespmc1.vub.ac.be/Books/Boulding.pdf>.

Boyd, Robert, and Peter J. Richerson. 1985. *Culture and the Evolutionary Process*. Chicago: University of Chicago Press.

Canzona. 2018. “Teilhard’s Legacy Can’t Be Reduced to Racism: A Response to John Slattery - Rewire News Group - Religion Dispatches.” *Rewire News Group*. August 22. <https://rewirenewsgroup.com/religion-dispatches/2018/08/22/teilhards-legacy-cant-be-reduced-to-racism-a-response-to-john-slattery/>.

Cave, Stephen. 2012. *Immortality: The Quest to Live Forever and How It Drives Civilization*. New York: Crown Publishers.

Christian, D. 2004. *Maps of Time: An Introduction to Big History*. University of California Press.

Ciccarelli, Francesca D., Tobias Doerks, Christian von Mering, Christopher J. Creevey, Berend Snel, and Peer Bork. 2006. “Toward Automatic Reconstruction of a Highly Resolved Tree of Life.” *Science* 311 (5765): 1283–87. doi:10.1126/science.1123061.

Clark, Andy. 2003. *Natural-Born Cyborgs: Minds, Technologies, and the Future of Human Intelligence*. Oxford University Press.

Clark, Andy, and David Chalmers. 1998. “The Extended Mind.” *Analysis* 58 (1): 7–19. doi:10.1093/analys/58.1.7. <http://www.jstor.org/stable/3328150>.

Conant, R. C, and W. Ross Ashby. 1970. “Every Good Regulator of a System Must Be a Model of That System.” *International Journal of Systems Science* 1 (2). Taylor & Francis: 89–97. doi:10.1080/00207727008920220. pespmc1.vub.ac.be/books/Conant_Ashby.pdf.

Darwin, C. 1887. *The Autobiography of Charles Darwin*. Barnes & NoblePublishing (2005).

Dawkins, Richard. 1995. *River out of Eden: A Darwinian View of Life*. Basic Books.

de Rosnay, J. 1979. *The Macroscope: A New World Scientific System*. HarperCollins Publishers. <https://www.xn--7dbl2a.com/wp-content/uploads/1978/05/THE_MACROSCOPE.pdf>.

———. 1988. *Le Cerveau Planétaire*. Points 193. Paris: Seuil.

———. 2000. *The Symbiotic Man: A New Understanding of the Organization of Life and a Vision of the Future*. McGraw-Hill Companies.

———. 2012. *Surfer La Vie : Vers La Société Fluide*. Les liens qui liberent editions.

Deacon, Terrence William. 1998. *The Symbolic Species: The Co-Evolution of Language and the Brain*. 202. WW Norton & Company.

Dehaene, S. 2014. *Consciousness and the Brain: Deciphering How the Brain Codes Our Thoughts*. Penguin Books.

Dehaene, S., M. Kerszberg, and J.-P. Changeux. 1998. “A Neuronal Model of a Global Workspace in Effortful Cognitive Tasks.” *Proceedings of the National Academy of Sciences* 95 (24): 14529–34. doi:10.1073/pnas.95.24.14529. <http://www.pnas.org/cgi/doi/10.1073/pnas.95.24.14529>.

Delahaye, J. P., and C. Vidal. 2019. “Universal Ethics: Organized Complexity as an Intrinsic Value.” In *Evolution, Development and Complexity: Multiscale Evolutionary Models of Complex Adaptive Systems*, edited by Georgi Yordanov Georgiev, Claudio Flores Martinez, Michael E. Price, and John M. Smart. Springer. doi:10.5281/zenodo.1172976. <https://zenodo.org/record/1285656>.

Deutsch, David. 1997. *The Fabric of Reality the Science of Parallel Universes- and Its Implications*. New York: Penguin Books.

Ekman, Dr Paul. 2014. *Moving Toward Global Compassion*. San Francisco: Paul Ekman Group.

Gillings, Michael R., Martin Hilbert, and Darrell J. Kemp. 2016. “Information in the Biosphere: Biological and Digital Worlds.” *Trends in Ecology & Evolution* 31 (3): 180–89. doi:10.1016/j.tree.2015.12.013.

Gini, Corrado. 1927. “The Scientific Basis of Fascism.” *Political Science Quarterly* 42 (1): 99. doi:10.2307/2142862. <http://www.jstor.org/stable/2142862?origin=crossref>.

Glick, Thomas. 2008. “Miquel Crusafont, Teilhard de Chardin and the Reception of the Synthetic Theory in Spain.” In *The Reception of Charles Darwin in Europe*, edited by Eve-Marie Engels and Thomas Glick. A&C Black.

Goertzel, Ben. 2010. *A Cosmist Manifesto: Practical Philosophy for the Posthuman Age*. Humanity+. <http://goertzel.org/CosmistManifesto_July2010.pdf>.

Harnad, Steven. 1990. “Scholarly Skywriting and the Prepublication Continuum of Scientific Inquiry.” *Psychological Science* 1 (6). SAGE Publications Inc: 342–44. doi:10.1111/j.1467-9280.1990.tb00234.x.

Haught, John F. 2019. “Trashing Teilhard | Commonweal Magazine.” February 12. <https://www.commonwealmagazine.org/trashing-teilhard>.

Helbing, D., S. Bishop, R. Conte, P. Lukowicz, and J. B. McCarthy. 2012. “FuturICT: Participatory Computing to Understand and Manage Our Complex World in a More Sustainable and Resilient Way.” *The European Physical Journal Special Topics* 214 (1): 11–39. doi:10.1140/epjst/e2012-01686-y. <http://www.springerlink.com/index/10.1140/epjst/e2012-01686-y>.

Hesse, M. 1966. *Models and Analogies in Science*. Notre Dame, IN: Notre Dame University Press.

Heylighen, F. 1999a. “Collective Intelligence and Its Implementation on the Web: Algorithms to Develop a Collective Mental Map.” *Computational & Mathematical Organization Theory* 5 (3): 253–80. <http://link.springer.com/article/10.1023/A:1009690407292>.

———. 1999b. “The Growth of Structural and Functional Complexity During Evolution.” In *The Evolution of Complexity: The Violet Book of “Einstein Meets Magritte*, edited by F. Heylighen, Johan Bollen, and A. Riegler, 17–44. Dordrecht: Kluwer Academic Publishers. <http://pespmc1.vub.ac.be/Papers/ComplexityGrowth.pdf>.

———. 2002. “The Global Brain as a New Utopia.” *Zukunftsfiguren, Suhrkamp, Frankurt*. <http://pespmc1.vub.ac.be/Papers/GB-Utopia.pdf>.

———. 2005. “Conceptions of a Global Brain: An Historical Review.” *Technological Forecasting and Social Change*. <http://pespmc1.vub.ac.be/Papers/GBconceptions.pdf>.

———. 2007a. “Accelerating Socio-Technological Evolution: From Ephemeralization and Stigmergy to the Global Brain.” In *Globalization as an Evolutionary Process: Modeling Global Change*, edited by George Modelski, Tessaleno Devezas, and William Thompson, Routledge, 286–335. London. <http://pespmc1.vub.ac.be/Papers/AcceleratingEvolution.pdf>.

———. 2007b. “The Global Superorganism: An Evolutionary-Cybernetic Model of the Emerging Network Society.” *Social Evolution & History* 6 (1): 58–119. doi:10.1.1.43.3443. <http://pespmc1.vub.ac.be/papers/Superorganism.pdf>.

———. 2015. “Return to Eden? Promises and Perils on the Road to a Global Superintelligence.” In *The End of the Beginning: Life, Society and Economy on the Brink of the Singularity*, edited by Ben Goertzel and Ted Goertzel. <http://pespmc1.vub.ac.be/Papers/BrinkofSingularity.pdf>.

Heylighen, F., and C. Joslyn. 2001. “Cybernetics and Second Order Cybernetics.” *Encyclopedia of Physical Science & Technology* 4: 155–70. <http://pespmc1.vub.ac.be/Papers/Cybernetics-EPST.pdf>.

Hitler, Adolf. 1939. *Mein Kampf*. Translated by James Vincent Murphy. Unexpurgated ed. London: Hurst and Blackett.

Hobbes, Thomas. 2011. *Leviathan*. Peterborough, Ont.: Broadview Press.

Holyoak, K. J., and P. Thagard. 1995. *Mental Leaps: Analogy in Creative Thought*. MIT Press.

Kardashev, N. S. 1964. “Transmission of Information by Extraterrestrial Civilizations.” *Soviet Astronomy* 8 (2): 217–20. <http://adsabs.harvard.edu/abs/1964SvA.....8..217K>.

Kelly, Kevin. 2010. *What Technology Wants*. New York: Viking.

———. 2014. “Over the Long Term, the Future Is Decided by Optimists.” *Twitter*. <https://twitter.com/kevin2kelly/status/459723553642778624>.

Keynes, John Maynard. 1932. *Essays in Persuasion*. New York: Harcourt, Brace and Company.

Koltko-Rivera, Mark E. 2004. “The Psychology of Worldviews.” *Review of General Psychology* 8 (1): 3–58. doi:10.1037/1089-2680.8.1.3. <https://www.academia.edu/3089027/The_psychology_of_worldviews>.

Korycansky, D. G., Gregory Laughlin, and Fred C. Adams. 2001. “Astronomical Engineering: A Strategy For Modifying Planetary Orbits.” *Astrophysics and Space Science* 275 (4): 349–66. doi:10.1023/A:1002790227314. <http://arxiv.org/abs/astro-ph/0102126>.

Landauer, R. 1961. “Irreversibility and Heat Generation in the Computing Process.” *IBM Journal of Research and Development* 5 (3): 183–91. doi:10.1147/rd.53.0183.

Last, Cadell. 2015. “Human Metasystem Transition (HMST) Theory.” *Journal of Evolution and Technology* 25 (1): 1–16.

Lem, Stanisław. 2013. *Summa Technologiae*. Electronic Mediations 40. Minneapolis: University of Minnesota Press.

Lloyd, S. 2000. “Ultimate Physical Limits to Computation.” *Nature* 406: 1047–54. <https://arxiv.org/abs/quant-ph/9908043>.

Lovelock, James. 1979. *Gaia, a New Look at Life on Earth*. Oxford ; New York: Oxford University Press.

Magee, Christopher L. 2012. “Towards Quantification of the Role of Materials Innovation in Overall Technological Development.” *Complexity* 18 (1): 10–25. doi:10.1002/cplx.20309. <http://doi.wiley.com/10.1002/cplx.20309>.

Mayer-Kress, G., and C. Barczys. 1995. “The Global Brain as an Emergent Structure from the Worldwide Computing Network, and Its Implications for Modelling.” *The Information Society* 11 (1): 1–28.

Maynard Smith, John, and Eörs Szathmáry. 1995. *The Major Transitions in Evolution*. Oxford ; New York: W.H. Freeman Spektrum.

Mayr, Ernst, and William B. Provine. 1998. *The Evolutionary Synthesis: Perspectives on the Unification of Biology*. Harvard University Press.

Miller, James Grier. 1978. *Living Systems*. McGraw-Hill New York. <https://archive.org/details/LivingSystems>.

Minsky, M. 1986. *The Society of Mind*. Simon & Schuster, Inc. New York, NY, USA.

Montes, Gabriel Axel, and Ben Goertzel. 2019. “Mindplexes, Non-Ordinary Consciousness, and Artificial General Intelligence.” Preprint. MindRxiv. doi:10.31231/osf.io/nwyts. <https://osf.io/nwyts>.

Moravec, Hans P. 1988. *Mind Children: The Future of Robot and Human Intelligence*. Cambridge, Mass: Harvard University Press.

Musk, Elon. 2019. “An Integrated Brain-Machine Interface Platform with Thousands of Channels.” *BioRxiv*, August. Cold Spring Harbor Laboratory, 703801. doi:10.1101/703801. <https://www.biorxiv.org/content/10.1101/703801v4>.

Niele, Frank. 2005. *Energy Engine of Evolution*. Amsterdam; Kidlington: Elsevier.

Odum, Eugene P. 2001. “The ‘Techno-Ecosystem.’” *Bulletin of the Ecological Society of America* 82 (2): 137–38.

Otlet, P. 1935. *Monde: Essai d’Universalisme*. Brussels: Mundaneum.

Parunak, H. V. D. 2006. “A Survey of Environments and Mechanisms for Human-Human Stigmergy.” *Environments for Multi-Agent Systems II*, 163–86. <http://citeseerx.ist.psu.edu/viewdoc/download?doi=10.1.1.100.6974&rep=rep1&type=pdf>.

Phillips, D. C. 1970. “Organicism in the Late Nineteenth and Early Twentieth Centuries.” *Journal of the History of Ideas* 31 (3). University of Pennsylvania Press: 413–32. doi:10.2307/2708514. <https://www.jstor.org/stable/2708514>.

Pinker, Steven. 2011. *The Better Angels of Our Nature: Why Violence Has Declined*. Viking Adult.

Pirie, Madsen. 2006. *How to Win Every Argument: The Use and Abuse of Logic*. London ; New York: Continuum.

Ptolemy, Robert Barry. 2011. *Transcendent Man*. Documentary. Ptolemaic Productions, Therapy Content.

Ree, Erik van. 1993. “Stalin’s Organic Theory of the Party.” *The Russian Review* 52 (1). [Wiley, Editors and Board of Trustees of the Russian Review]: 43–57. doi:10.2307/130857. <https://www.jstor.org/stable/130857>.

Rescher, N. 2006. *Philosophical Dialectics: An Essay on Metaphilosophy*. State University of New York Press.

Richerson, Peter, and Robert Boyd. 2006. *Not by Genes Alone: How Culture Transformed Human Evolution*. University Of Chicago Press.

Rifkin, Jeremy. 2014. *The Zero Marginal Cost Society: The Internet of Things, the Collaborative Commons, and the Eclipse of Capitalism*. St. Martin’s Publishing Group.

Rousseau, Jean-Jacques. 2002. *The Social Contract*. Translated by Gita May. Yale University Press.

Russell, P. 1982. *The Awakening Earth: The Global Brain*.

———. 1995. *Global Brain Awakens: Our Next Evolutionary Leap*. Peter Russell.

Sagan, Carl. 1973. *The Cosmic Connection; an Extraterrestrial Perspective*. 1^st^ ed. Garden City, N.Y: Anchor Press.

———. 1995. *The Demon-Haunted World: Science as a Candle in the Dark*. 1^st^ ed. New York: Random House.

Shoshitaishvili, Boris. 2020. “Deep Time and Compressed Time in the Anthropocene: The New Timescape and the Value of Cosmic Storytelling:” *The Anthropocene Review*, April. SAGE PublicationsSage UK: London, England. doi:10.1177/2053019620917707.

Slattery, John P. 2017. “Dangerous Tendencies of Cosmic Theology: The Untold Legacy of Teilhard de Chardin.” *Philosophy and Theology* 29 (1): 69–82.

———. 2018a. “Pierre Teilhard de Chardin’s Legacy of Eugenics and Racism Can’t Be Ignored.” *Religion Dispatches*. May 21. <https://religiondispatches.org/pierre-teilhard-de-chardins-legacy-of-eugenics-and-racism-cant-be-ignored/>.

———. 2018b. “Author Responds to Criticism of Teilhard Eugenics Essay.” *Religion Dispatches*. August 22. <https://religiondispatches.org/author-responds-to-criticism-of-teilhard-eugenics-essay/>.

———. 2019. “Teilhard & Eugenics.” *Commonweal Magazine*, March 12. <https://www.commonwealmagazine.org/teilhard-eugenics>.

Smart, J. M. 2009. “Evo Devo Universe? A Framework for Speculations on Cosmic Culture.” In *Cosmos and Culture: Cultural Evolution in a Cosmic Context*, edited by S. J. Dick and Mark L. Lupisella, 201–95. Washington D.C.: Government Printing Office, NASA SP-2009-4802. <http://accelerating.org/downloads/SmartEvoDevoUniv2008.pdf>.

———. 2012. “The Transcension Hypothesis: Sufficiently Advanced Civilizations Invariably Leave Our Universe, and Implications for METI and SETI.” *Acta Astronautica* 78 (September): 55–68. doi:10.1016/j.actaastro.2011.11.006. <http://accelerating.org/articles/transcensionhypothesis.html>.

Spencer, H. 1895. *The Principles of Sociology*. D. Appleton.

Spier, F. 1996. *The Structure of Big History from the Big Bang Until Today*. Amsterdam University Press.

Steinhart, Eric. 2008. “Teilhard de Chardin and Transhumanism.” *Journal of Evolution and Technology* 20 (1): 1–22. <http://jetpress.org/v20/steinhart.htm>.

Stewart, John E. 2000. *Evolution’s Arrow: The Direction of Evolution and the Future of Humanity*. Chapman Press. <http://users.tpg.com.au/users/jes999/EvAr.pdf>.

———. 2001. “Future Psychological Evolution.” *Dynamical Psychology* 2001. <http://cogprints.org/1995/>.

———. 2010. “The Meaning of Life in a Developing Universe.” *Foundations of Science* 15 (4): 395–409. doi:10.1007/s10699-010-9184-9. <http://cogprints.org/6655/>.

Stock, G. 1993. *Metaman: The Merging of Humans and Machines Into a Global Superorganism*. Simon & Schuster.

Swimme, Brian, and Thomas Berry. 1992. *The Universe Story: From the Primordial Flaring Forth to the Ecozoic Era - a Celebration of the Unfolding of the Cosmos*. San Francisco, Calif.: HarperSan Francisco.

Szathmáry, Eörs. 2015. “Toward Major Evolutionary Transitions Theory 2.0.” *Proceedings of the National Academy of Sciences* 112 (33): 10104–11.

Teilhard de Chardin, Pierre. 1945. “Christianisme et Évolution.” In *Comment je crois*. Vol. 10. Seuil. <http://dx.doi.org/doi:10.1522/030432822>.

———. 1947. “Un Colloque Scientifique Sur l’Evolution.” *Etudes*, no. May: 257–59.

———. 1959a. “The Formation of the Noosphere: A Plausible Biological Interpretation of Human History.” In *The Future of Man*, 149–78. New York: Image Books Doubleday. <https://www.organism.earth/library/document/formation-of-the-noosphere>.

———. 1959b. *The Phenomenon of Man*. New York: Harper.

Theraulaz, G., and E. Bonabeau. 1999. “A Brief History of Stigmergy.” *Artificial Life* 5 (2): 97–116. doi:10.1162/106454699568700. <http://dx.doi.org/10.1162/106454699568700>.

Tipler, F. J. 1995. *The Physics of Immortality*. Doubleday.

Tononi, Giulio, and Gerald M. Edelman. 1998. “Consciousness and Complexity.” *Science* 282 (5395): 1846–51. <http://science.sciencemag.org/content/282/5395/1846.short>.

Turchin, V. 1977. *The Phenomenon of Science*. New York: Columbia University Press. <http://pespmc1.vub.ac.be/POS/TurPOS.pdf>.

———. 1995. “A Dialogue on Metasystem Transition.” *World Futures* 45: 5–57. <http://en.scientificcommons.org/42814465>.

Tyrrell, Toby. 2013. *On Gaia: A Critical Investigation of the Relationship between Life and Earth*. Princeton University Press.

UNESCO, Jake Lewis, and Tiffany Straza. 2021. “UNESCO Science Report: The Race against Time for Smarter Development; Executive Summary.” SC-2021/WS/7. UNESCO. <https://unesdoc.unesco.org/ark:/48223/pf0000377250>.

Vernadsky, V. I. 1926. *The Biosphere*. New York: Copernicus, 1998.

———. 1945. “The Biosphere and the Noösphere.” *American Scientist* 33 (1): xxii–12. doi:10.2307/27826043. <http://www.jstor.org/stable/27826043>.

———. 2012. *The Biosphere*. Springer Science & Business Media.

Vidal, C. 2011. “Black Holes: Attractors for Intelligence?” In Buckinghamshire, Kavli Royal Society International Centre. <http://arxiv.org/abs/1104.4362>.

———. 2014a. “Cosmological Ethics and Immortality.” In *The Beginning and the End: The Meaning of Life in a Cosmological Perspective*. New York: Springer. <http://arxiv.org/abs/1301.1648>.

———. 2014b. *The Beginning and the End - The Meaning of Life in a Cosmological Perspective*. Frontiers Collection. Springer. <http://arxiv.org/abs/1301.1648>.

———. 2015. “Distributing Cognition: From Local Brains to the Global Brain.” In *The End of the Beginning: Life, Society and Economy on the Brink of the Singularity*, edited by B. Goertzel and T. Goertzel, 325–72. Humanity+ Press. <http://tinyurl.com/vidal2015>.

———. 2016. “Stellivore Extraterrestrials? Binary Stars as Living Systems.” *Acta Astronautica* 128: 251–56. doi:10.1016/j.actaastro.2016.06.038. <https://zenodo.org/record/164853>.

———. 2020. “Energy Rate Density as a Technosignature: The Case for Stellivores.” *Presented at Life in the Universe: Big History, SETI and the Future of Humankind. IBHA & INAF-IASF MI Symposium 15-16 July 2019*. <http://www.clemvidal.com/s/Vidal-2020-ERD-as-a-technosignature-case-for-stellivores.pdf>.

———. 2021. “Pierre Teilhard de Chardin: A Visionary in Controversy”, *History and Philosophy of Life Sciences*, https:// doi.org/10.1007/s40656-021-00475-7.

Vidal, C., F. Heylighen, and D. Weinbaum. 2013. “Evolutionary Psychology of the Global Brain and FuturICT.” European Conference on Complex Systems presented at the Satellite Meeting “Global Computing for our Complex Hyper-connected World,” Barcelona, September 19. <http://prezi.com/pv9t6-ndmyy6/eccs-2013-evolutionary-psychology-of-the-global-brain-and-futurict/>.

Weinberg, S. 1993. *The First Three Minutes: A Modern View of the Origin of the Universe*. Basic Books.

Wells, H. G. 1938. *World Brain*. Doubleday, Doran & co., inc.

West, Stuart A., Roberta M. Fisher, Andy Gardner, and E. Toby Kiers. 2015. “Major Evolutionary Transitions in Individuality.” *Proceedings of the National Academy of Sciences* 112 (33): 10112–19.

Wildiers, N. M. 1960. *Teilhard de Chardin*. Classiques Du XXe Siècle 36. Paris: Éditions universitaires.

Wilson, David Sloan. 1983. “The Group Selection Controversy: History and Current Status.” *Annual Review of Ecology and Systematics* 14 (1). Annual Reviews 4139 El Camino Way, PO Box 10139, Palo Alto, CA 94303-0139, USA: 159–87.

———. 2019. *This View of Life: Completing the Darwinian Revolution*. New York: Pantheon.

Young, George M. 2012. *The Russian Cosmists: The Esoteric Futurism of Nikolai Fedorov and His Followers*. Oxford ; New York: Oxford University Press.
